# Immune infiltration-related N6-methyladenosine RNA methylation regulators influence the malignancy and prognosis of endometrial cancer

**DOI:** 10.18632/aging.203157

**Published:** 2021-06-16

**Authors:** Jian Ma, Di Yang, Xiao-Xin Ma

**Affiliations:** 1 Department of Obstetrics and Gynecology, Shengjing Hospital of China Medical University, Shenyang 110004, China

**Keywords:** endometrial cancer, N6-methyladenosine, immune infiltration, ZC3H13, YTHDC1

## Abstract

N6-methyladenosine (m6A) RNA methylation is associated with malignant tumor progression and is modulated by various m6A RNA methylation regulator proteins. However, its role in endometrial cancer is unclear. In this work, we analyzed sequence, copy number variation, and clinical data obtained from the TCGA database. Expression was validated using real-time quantitative polymerase chain reaction and immunohistochemistry. Changes in m6A RNA methylation regulators were closely related to the clinicopathological stage and prognosis of endometrial cancer. In particular, ZC3H13, YTHDC1, and METTL14 were identified as potential markers for endometrial cancer diagnosis and prognosis. The TIMER algorithm indicated that immune cell infiltration correlated with changes in *ZC3H13, YTHDC1*, and *METTL14* expression. Meanwhile, *ZC3H13* or *YTHDC1* knockdown promoted the proliferation and invasion of endometrial cancer cells. Through gene enrichment analysis, we constructed a regulatory network in order to explore the potential molecular mechanism involving *ZC3H13*, *YTHDC1*, and *METTL14*. Virtual screening predicted interactions of potential therapeutic compounds with METTL14 and YTHDC1. These findings advance the understanding of RNA epigenetic modifications in endometrial cancer while identifying m6A regulators associated with immune infiltration, prognosis, and potential treatment strategies.

## INTRODUCTION

Globally, endometrial cancer (EC) is the second most common gynecological malignancy after cervical cancer. Meanwhile, in European and American countries, EC ranks first among gynecological malignancies in terms of incidence [1]. Although the overall prognosis for EC is favorable, the continuous increase in associated morbidity and mortality rates makes prevention and control increasingly challenging, with surgical interventions currently employed as the first line of treatment [2]. Clinically, patients with EC are often stratified based on postoperative pathological results that guide follow-up treatment [3]. EC is a heterogeneous disease with widely variable clinical outcomes, both in terms of prognosis and treatment response. With the advent of molecular characterization technologies, EC has been divided into four molecular categories, namely POLE ultra-mutated, microsatellite-instable (MSI), copy-number low/microsatellite-stable (MSS), and copy number high/serous-like [4]. Tumors of the POLE subtype often harbor *POLE* (100%), *PTEN* (94%), *PIK3CA* (71%), *PIK3R1* (65%), and *KRAS* (53%) mutations. Those of the MSI subtype often carry mutated *PIK3CA* (54%), *PIK3R1* (42%), and *PTEN* (88%). CNL/MSS subtypes exhibit mutations in *PTEN* (77%) and *PIK3CA* (53%), whereas CNH subtypes harbor mutated *TP53* (92%), *FBXW7* (22%), *PPP2R1A* (22%), *PTEN* (10%), and *PIK3CA* (47%). Overall, molecular genotyping has demonstrated superior reproducibility compared to tissue typing and thus offers greater clinical value than simple morphological classification [5–6].

N6-methyladenosine (m6A) methylation is a common modification of eukaryotic mRNA. Further, m6A regulator proteins participate in the modification of various RNA types, including transfer RNA (tRNA) and ribosomal RNA (rRNA) [7]. The cellular m6A status is regulated by groups of proteins called “writers” (WTAP, METTL3, and METTL14), “erasers” (FTO and ALKBH5), and “readers” (YTHDF1, YTHDF2, YTHDF3, YTHDC1, YTHDC2, HNRNPC, IGF2BP1, IGF2BP2, IGF2BP3) [8–10]. These regulatory factors modulate the stability, splicing, intracellular distribution, and translational changes of mRNA by adding, removing, or reading m6A modifications [11]. As a potential tumor biomarker, m6A plays roles in various biological processes within cancer [12]. The dysregulation of m6A is related to the occurrence and development of various malignancies. Further, changes in m6A-modifying enzyme levels affect the expression of downstream oncogenes or tumor suppressor genes by altering mRNA methylation [13]. Although RNA methylation and demethylation are reversible processes, the structure of RNA is highly conserved, suggesting that RNA epigenetic modifications may represent novel targets for cancer precision medicine [14]. For instance, promoting or inhibiting the key m6A regulators may interfere with tumor development.

Immunotherapy has revolutionized cancer treatment, with several treatment types, including adoptive cell transfer and immune checkpoint inhibitors, exhibiting durable clinical responses [15]. Hence, immunotherapy represents potential treatment options for patients with advanced EC. However, only a limited number of patients benefit from the currently available regimens [16]. This may be due to differences in tumor-infiltrating immune cells within the tumor microenvironment, which affect the clinical outcome in cancer patients by regulating immune escape [17, 18]. In pancreatic and colorectal cancer, the expression of m6A regulators is related to tumor immune cell infiltration, providing certain cues for the therapeutic response of ICIS [19, 20]. Therefore, exploring m6A regulatory factors implicated in immune cell infiltration may reveal new strategies for EC treatment.

In this study, we aimed to analyze clinical, sequencing, and copy number variation (CNV) data in EC in relation to m6A regulators. We used TCGA data to evaluate the relationship between m6A regulator gene changes, clinicopathological characteristics, and survival rates. The TIMER algorithm indicated that the expression of *METTL14*, *ZC3H13,* and *YTHDC1* was positively correlated with immune cell infiltration levels. *In vitro* experiments revealed that ZC3H13 and YTHDC1 knockdown promoted the proliferation and invasion of EC cells. Finally, we used virtual screening to investigate which pharmacological compounds preferentially interacted with METTL14 or YTHDC1. The current findings highlight potential biomarkers and therapeutic targets for EC.

## RESULTS

### m6A regulator gene mutations and copy number variation in endometrial cancer patients

**Figure 1:**
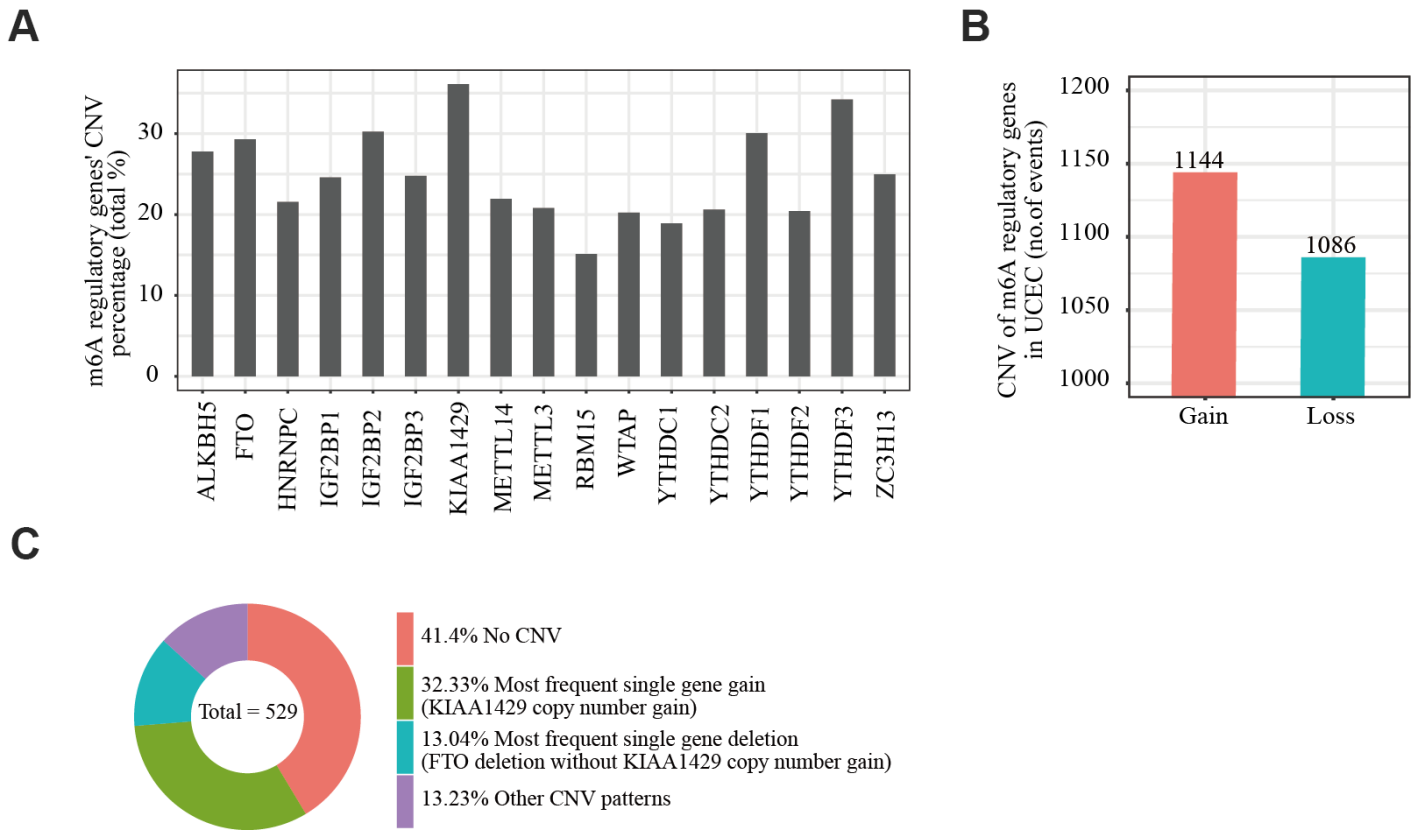
**Copy number variation of m6A regulatory genes in endometrial cancer.** (**A**) Analysis of the percentage of CNVs among m6A regulatory genes in TCGA data of 529 EC samples. (**B**) Incidence of m6A regulatory gene copy number increase or loss in EC samples. (**C**) The most common CNV among m6A regulatory genes in EC samples.

We obtained somatic mutation and CNV data of EC patients from the UCSC Xena database and analyzed changes in m6A regulatory genes. Among the EC samples from 433 cases, mutations in m6A regulatory genes were detected in 133 independent samples (Supplementary Table 4). Of 529 EC samples with CNV data, CNVs were frequently observed in 17 m6A regulator-encoding genes (Figure 1A). Among these, the frequency of CNV was highest in the m6A writer gene *KIAA1429* (36.11%, 191/529, Table 1), followed by reader gene *YTHDF3* (34.22%, 181/529, Table 1). We then evaluated the CNV pattern in EC samples and found that most CNV events were increases in copy number (1144/1086; Figure 1B). Finally, we determined the most common CNV type in m6A regulatory genes. Copy number gain of *KIAA1429* was the most frequent alteration among all m6A regulatory gene CNVs (Figure 1C).

**Table 1:** Different CNV patterns occur in endometrial cancer samples (n=529).

		**Diploid**	**Amplification**	**Copy number gain**	**Deep deletion**	**Shallow deletion**	**CNV sum**	**Percentage (%)**
Eraser	ALKBH5	382	5	22	4	116	147	27.79
	FTO	374	5	14	3	133	155	29.30
Writer	METTL3	419	3	51	1	55	110	20.79
	METTL14	413	1	8	3	104	116	21.93
	WTAP	422	3	59	1	44	107	20.23
	KIAA1429	338	21	150	0	20	191	36.11
	RBM15	449	4	42	1	33	80	15.12
	ZC3H13	397	1	30	1	100	132	24.95
Reader	YTHDC1	429	3	12	1	84	100	18.90
	YTHDC2	420	0	16	2	91	109	20.60
	YTHDF1	370	17	130	0	12	159	30.06
	YTHDF2	421	3	37	2	66	108	20.42
	YTHDF3	348	11	155	0	15	181	34.22
	HNRNPC	415	6	54	1	53	114	21.55
	IGF2BP1	399	5	46	1	78	130	24.57
	IGF2BP2	369	42	107	2	9	160	30.25
	IGF2BP3	398	11	70	2	48	131	24.76
		**Diploid**	**Amplification**	**Copy number gain**	**Deep deletion**	**Shallow deletion**	**CNV sum**	**Percentage (%)**

### m6A gene mutations are associated with endometrial cancer clinicopathological and molecular characteristics

We assessed the relationship between alterations (mutation and/or CNV) in m6A regulatory genes and clinicopathological characteristics in 433 EC patients. The alterations of m6A regulatory genes were related to clinical stage, grade, infiltration degree, and lymph node metastasis in patients (Table 2). Since *POLE, PTEN, PIK3CA, KRAS, PIK3R1, TP53, FBXW7,* and *PPP2R1A* play an important role in EC pathogenesis, we further assessed whether mutations in m6A regulators were related to the changes in these eight genes. Changes in m6A regulatory genes were significantly associated with *POLE, PTEN, KRAS, TP53*, and *PPP2R1A* alterations Next, we analyzed the influence of different CNV types on the mRNA expression of m6A regulatory genes. Indeed, expression was significantly correlated with different CNV patterns in 529 EC samples. For all 17 genes, the gene copy number increase (amplification) was related to enhanced mRNA expression, while shallow or deep deletions reduced mRNA expression (Supplementary Figure 1).

**Table 2:** Clinical pathological parameters and molecular characteristics of endometrial cancer patients with or without mutation/CNV of m6A regulatory genes.

		**With mutation or/and CNV**	**Without mutation and CNV**	***P***
Age	<=60	119	40	0.06834
	>60	224	47	
Clinical stage	I	191	72	**3.04E-05**
	II	37	5	
	III	94	10	
	IV	24	0	
Grade	G1	33	37	**2.20E-16**
	G2	62	28	
	G3	243	21	
	High Grade	8	1	
Infiltration degree	< 50	152	55	**2.17E-02**
	>= 50	136	26	
Lymphnode metastasis	Negative	120	40	0.06035
	Positive	207	42	
Distal metastasis	Negative	177	50	0.3215
	Positive	150	32	
POLE	WT	275	81	**0.004894**
	alteration	71	6	
PTEN	WT	151	8	**5.42E-09**
	alteration	195	79	
PIK3CA	WT	171	45	0.7918
	alteration	175	42	
KRAS	WT	293	58	**2.33E-04**
	alteration	53	29	
PIK3R1	WT	247	53	0.07808
	alteration	99	34	
TP53	WT	171	84	**3.70E-15**
	alteration	175	3	
FBXW7	WT	275	77	0.07572
	alteration	71	10	
PPP2R1A	WT	271	85	**4.73E-05**

Note: With mutation or CNV: Cases have mutant or CNV or mutant+CNV, confirmed through TCGA database. Without mutant and CNV: Cases with neither mutant nor CNV, confirmed through TCGA database.

### Correlation between m6A gene copy number variants and survival of endometrial cancer patients

**Figure 2:**
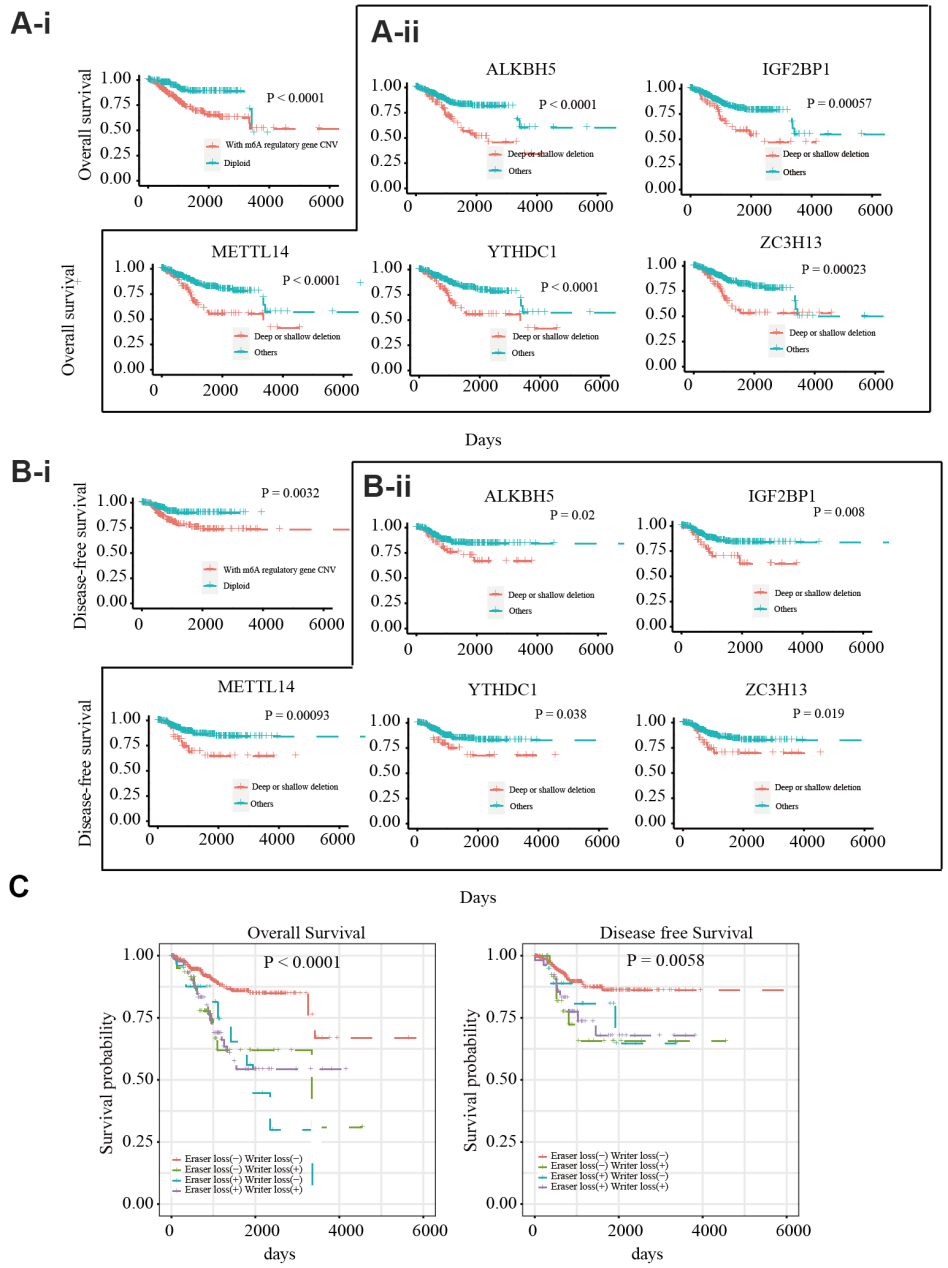
Relationship between copy number variants of m6A regulatory genes and OS/DFS in 412 EC patients (**A-i**) OS of EC patients with or without mutations in m6A regulatory genes*.* (**A-ii**) OS of EC patients with different CNV types for *IGF2BP1, ZC3H13, METTL14, ALKBH5,* and *YTHDC1.* (**B-i**) DFS of EC patients with or without mutations in m6A regulatory genes. (**B-ii**) DFS of EC patients with different CNV types for *IGF2BP1, ZC3H13, METTL14, ALKBH5,* and *YTHDC1.* (**C**) OS and DFS of EC patients with simultaneous alterations in writer and eraser genes.

To explore the prognostic value of CNVs in m6A regulatory genes, the effects of CNVs on the overall survival (OS) and disease-free survival (DFS) of 412 patients with EC were analyzed. Different CNV types in m6A regulatory genes were found to be related to OS and DFS (Figure 2A-i, Figure 2B-i). A separate analysis of the 17 genes revealed that patients with deep or shallow deletions of *IGF2BP1*, *ZC3H13*, *METTL14*, *ALKBH5*, or *YTHDC1* had lower OS and DFS than those who did not (Figure 2A-ii, Figure 2B-ii; and Supplementary Figures 2, 3). Univariate Cox analysis indicated that age, stage, grade, invasion, and lymph metastasis were independent risk factors for OS. The somatic mutation of *POLE, PTEN, PIK3CA, KRAS, PIK3R1, TP53, FBXW7,* and *PPP2R1A* genes were not significantly correlated with the survival rate of patients. Further, loss of eraser and writer genes was found to be an independent risk factor for DFS and OS (Table 3). Patients in the eraser loss (+) + and writer loss (+) groups had the shortest OS and DFS (Figure 2C). These results supported a relationship between decreased m6A regulator levels and poor survival.

**Table 3:** Univariate COX regression analysis of m6A regulatory genes for endometrial cancer patients' overall survival (OS) and disease-free survival (DFS).

**OS**					**DFS**			
**Variable**	**HR**	***P***	**Lower 95%CI**	**Upper 95%CI**	**HR**	***P***	**Lower 95% CI**	**Upper 95%CI**
Age	1.035	**0.001**	1.015	1.056	1.009	0.477	0.984	1.034
Stage	4.085	**0.001**	2.673	6.243	2.682	**0.001**	1.552	4.634
Grade	3.778	**0.001**	2.133	6.693	1.468	0.180	0.837	2.574
Invasion	1.012	**0.001**	1.008	1.016	1.009	0.079	0.999	1.018
Lymph Metastasis	0.973	**0.011**	0.952	0.994	1.006	0.589	0.983	1.030
PIK3CA	0.680	0.092	0.434	1.066	0.637	0.132	0.354	1.146
KRAS	0.571	0.111	0.286	1.138	0.640	0.304	0.274	1.498
POLE	0.640	0.184	0.331	1.237	0.511	0.153	0.203	1.283
TP53	1.326	0.198	0.863	2.037	1.407	0.229	0.807	2.453
PIK3R1	1.322	0.233	0.836	2.090	1.034	0.915	0.553	1.934
FBXW7	1.210	0.490	0.704	2.080	1.180	0.651	0.576	2.417
PTEN	0.867	0.507	0.568	1.323	0.880	0.642	0.513	1.509
PPP2R1A	0.981	0.950	0.544	1.771	0.351	0.078	0.109	1.125
W-E-	2.337	**0.0001**	1.533	3.564	1.831	**0.037**	1.036	3.236

Note: R-W-: m6A regulator alteration (Writer loss + Eraser loss); HR: (95% CI).

### m6A regulates the expression of key gene mRNAs in endometrial cancer patients

**Figure 3:**
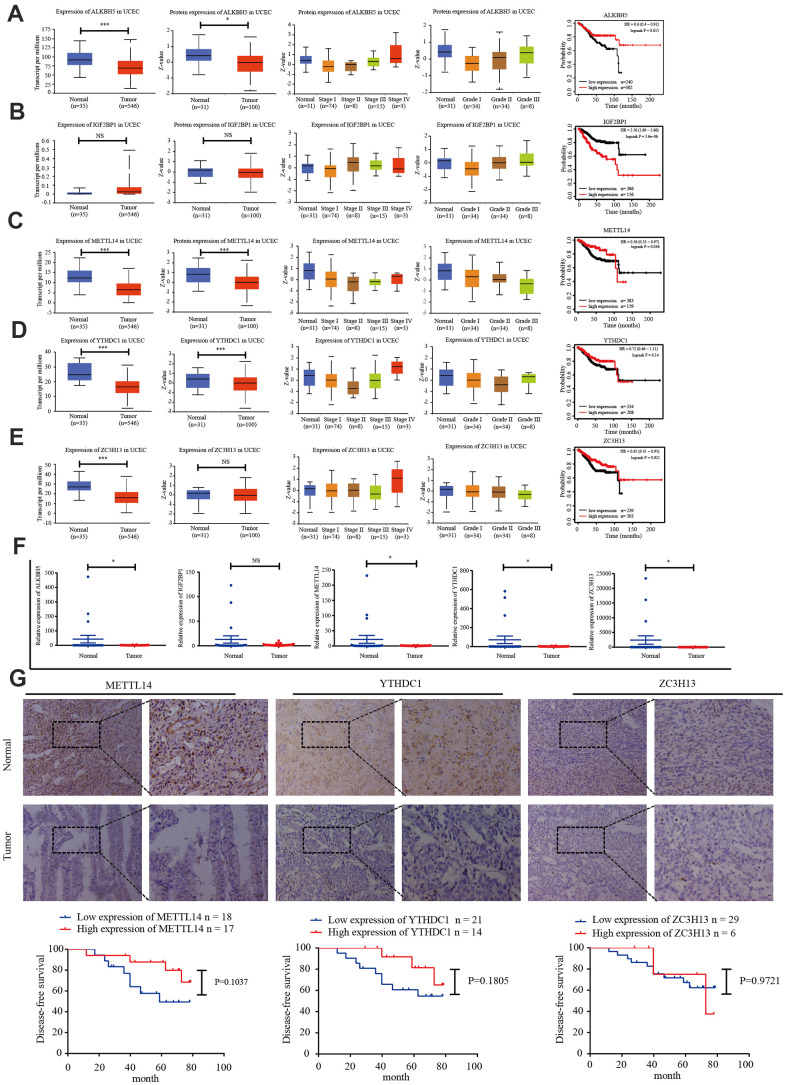
**Candidate m6A regulator protein and mRNA expression.** (**A**–**E**) TCGA and UALCAN website analysis of IGF2BP1, ZC3H13, METTL14, ALKBH5, and YTHDC1 mRNA expression in 35 normal and 546 EC tissues, protein expression in 31 normal and 100 EC tissues, and Kaplan-Meier survival curve of the prognostic signature based on five m6A-related genes. *P < 0.05, ** *P* < 0.01, *** *P* < 0.001. (**F**) Expression of the five genes in 34 EC tissues and 20 normal tissues, as determined by qRT-PCR. (**G**) Protein expression of ZC3H13, METTL14, and YTHDC1 in 35 EC tissues and 20 normal tissues was determined by immunohistochemistry. DFS curves for ZC3H13, METTL14, and YTHDC1 protein expression in 35 endometrial carcinoma cases. *P < 0.05, ** *P* < 0.01, *** *P* < 0.001.

TCGA data was used to analyze the expression of *IGF2BP1*, *ZC3H13*, *METTL14*, *ALKBH5*, and *YTHDC1* mRNA in 35 normal and 546 EC tissues. The UALCAN online tool (http://ualcan.path.uab.edu/index.html) was employed to assess corresponding protein expression in 31 normal and 100 EC tissues. ZC3H13, METTL14, ALKBH5, YTHDC1, and IGF2BP1 expression were downregulated. We performed univariate survival analysis of the EC TCGA data to determine the relationship between the expression of these five genes and EC prognosis. High expression of *IGF2BP1* was associated with unfavorable prognosis, while high expression of *ZC3H13*, *METTL14*, and *ALKBH5* was associated with improved prognosis (Figure 3A–3E).

We then detected the expression of *IGF2BP1, ZC3H13, METTL14, ALKBH5,* and *YTHDC1* in 20 normal endometrial tissues and 34 EC tissues via reverse transcription polymerase chain reaction (RT-PCR). *ZC3H13, METTL14,* and *YTHDC1* expression was lower in EC tissues (Figure 3F). The protein expression of ZC3H13, METTL14, and YTHDC1 was quantified in 20 normal endometrial tissues and 35 EC tissues using immunohistochemistry. All three proteins were primarily localized within the nucleus, and positive expression was indicated by brownish yellow or uniform brown granules. Moreover, the expression rates of ZC3H13, METTL14, and YTHDC1 in the EC group [17.1% (6/35); 48.6% (17/35); and 40.0% (14/35), respectively] were significantly lower than those in the normal control group [55.0% (11/20); 70.0% (14/20); and 60.0% (12/20), respectively; *P* < 0.05]. We then analyzed the association of ZC3H13, METTL14, and YTHDC1 protein expression with DFS of EC patients. High expression of ZC3H13, METTL14, and YTHDC1 correlated with a higher survival rate. However, these associations were not statistically significant (Figure 3G).

### Prognostic markers related to the immune microenvironment of endometrial cancer

**Figure 4:**
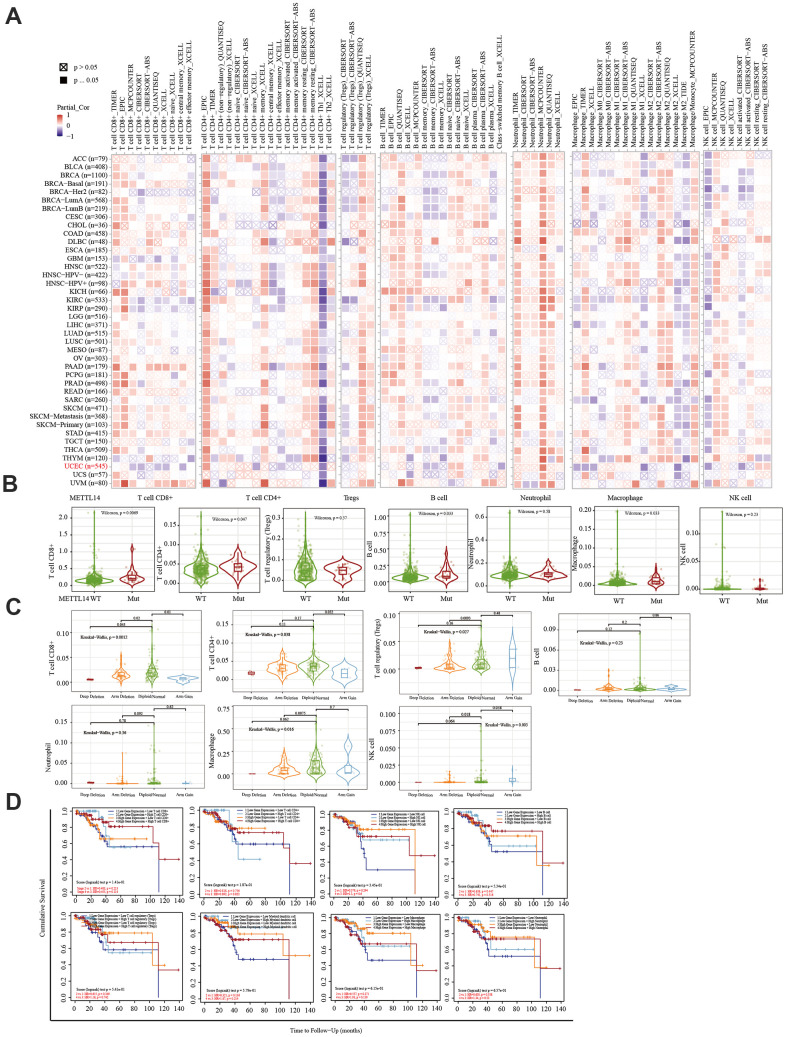
**Correlation between immune infiltration and the expression, mutation, SCNA status, and outcome module of METTL14.** (**A**) Heatmap depicting the correlation of METTL14 expression with six tumor-infiltrating immune cell types (CD4+ T cells, CD8+ T cells, Treg, B cells, neutrophils, macrophages, and NK cells) and the immune infiltration level in diverse cancer types. Spearman’s correlation was used for this analysis. (**B**) Violin plots to visualize the effect of *METTL14* gene mutations on immune cell infiltration and different infiltrating immune cell types in endometrial cancer. (**C**) Violin plots visualize the effect of *METTL14* SCNA, including ‘deep deletion’, ‘arm-level deletion’, ‘diploid/normal’, ‘arm-level gain’, and ‘high amplification’, on immune cell infiltration and different immune cell types in endometrial cancer, as determined by GISTIC2.0. (**D**) Outcome module showing the clinical stage relevance of tumor immune subsets as well as the hazard ratio and *P*-value for the Cox model. The log-rank *P*-value for the KM curve is shown on the KM curve plot.

**Figure 5:**
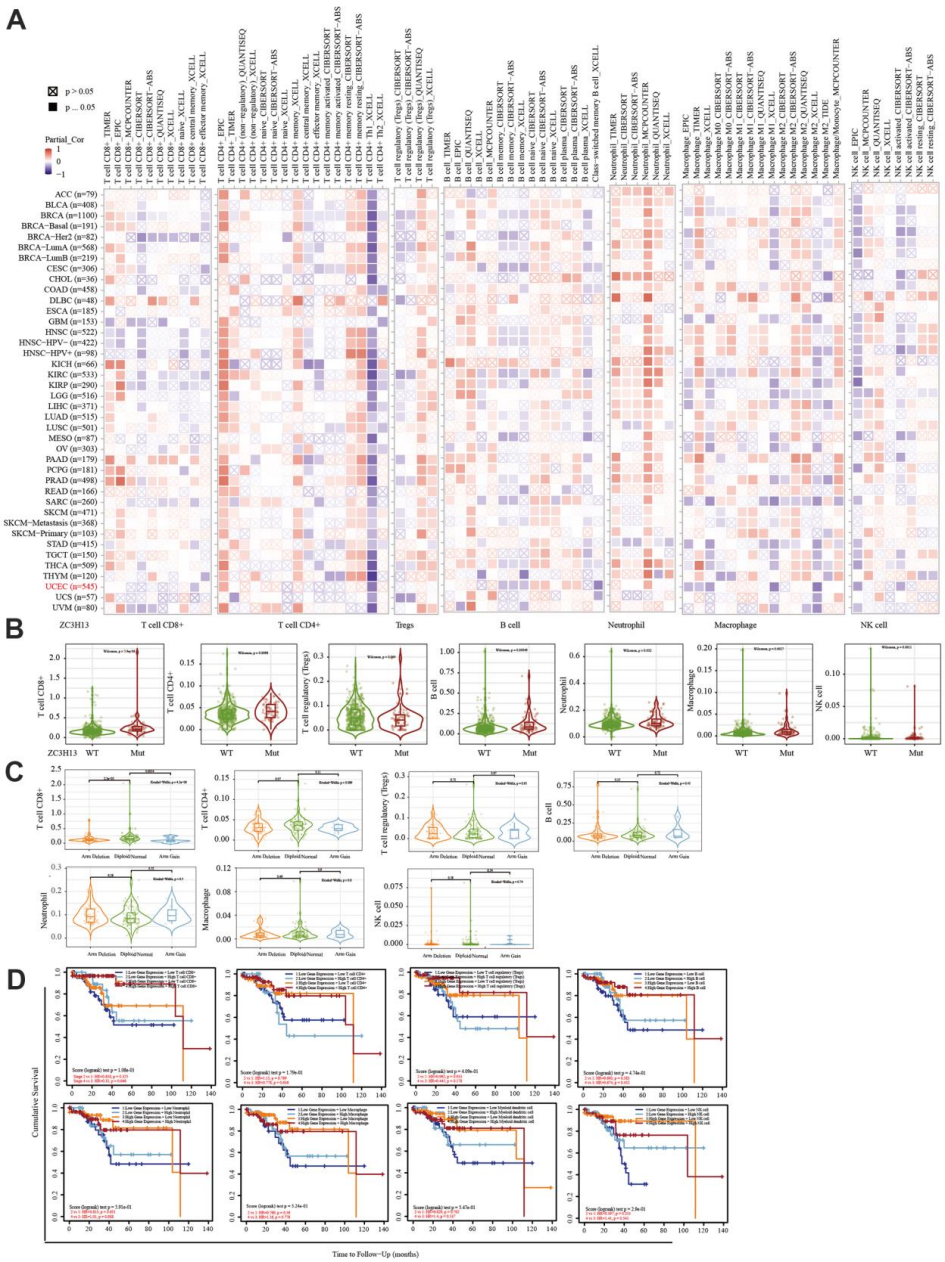
**Correlation between immune infiltration level and the expression, mutation, SCNA status, and outcome module of ZC3H13.** (**A**) Heatmap depicting the correlation of ZC3H13 expression with immune infiltration level in diverse cancer types. (**B**) Violin plots examining the effect of *ZC3H13* gene mutations on immune cell infiltration and immune cell types in endometrial cancer. (**C**) Violin plots depicting the effect of *ZC3H13* gene SCNA status on immune cell infiltration and immune cell types in endometrial cancer. (**D**) Outcome module showing the clinical relevance of tumor immune subsets.

**Figure 6:**
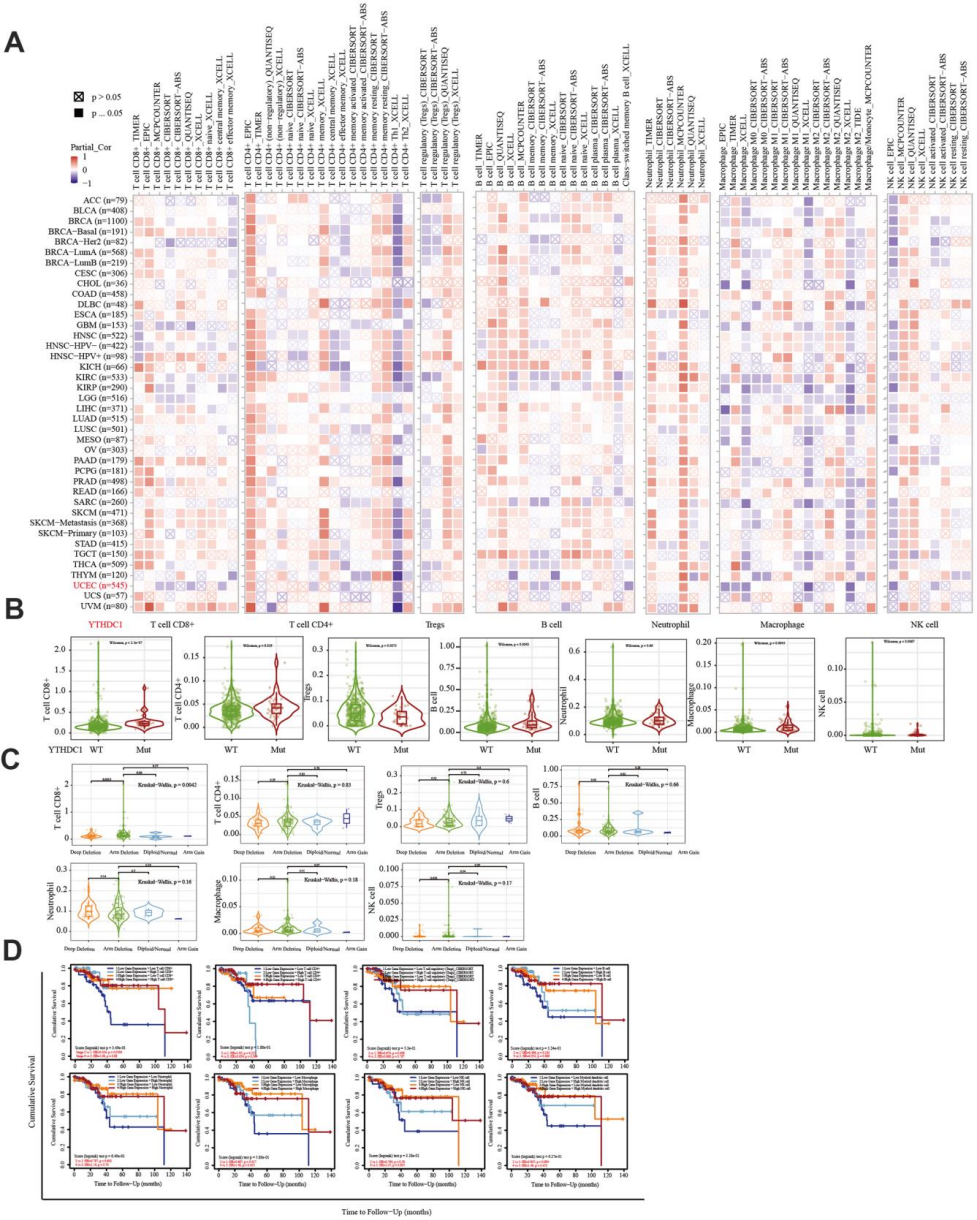
**Correlation between the immune infiltration level and the expression, mutation, SCNA status, and outcome module of YTHDC1.** (**A**) Heatmap depicting the correlation of YTHDC1 expression with the immune infiltration level in diverse cancer types. (**B**) Violin plots depicting the effect of *YTHDC1* gene mutations on immune cell infiltration and immune cell types in endometrial cancer. (**C**) Violin plots depicting the effect of *YTHDC1* SCNA status on immune cell infiltration and immune cell types in endometrial cancer. (**D**) Outcome module showing the clinical relevance of tumor immune subsets.

**Figure 7:**
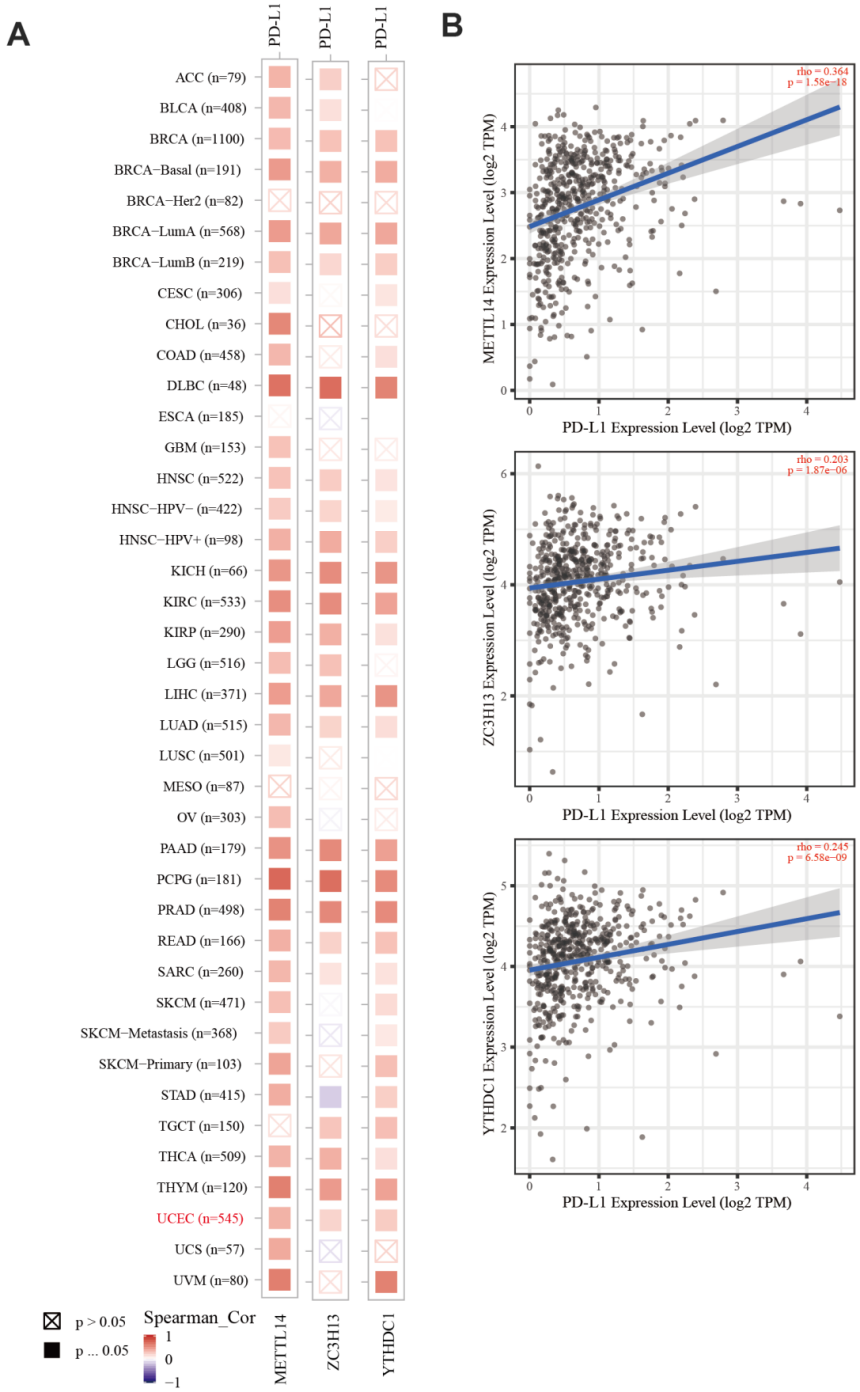
**The correlation of METTL14, ZC3H13, and YTHDC1 expression with PD-L1.** (**A**) Heatmap depicting the correlation of METTL14, ZC3H13, and YTHDC1 expression with PD-L1 in diverse cancer types. (**B**) Spearman’s correlation analysis was employed.

The TIMER database was used to analyze the correlation between EC immune infiltration level and the expression, mutation, and somatic copy number alterations (SCNAs) of *METTL14, ZC3H13,* and *YTHDC1* in 545 EC tissues. *METTL14, ZC3H13*, and *YTHDC1* expression were significantly related to immune cell infiltration in various tumors. In EC, the expression of *METTL14* was positively correlated with CD4+ T cell infiltration and negatively correlated with macrophage infiltration (Figure 4A). *ZC3H13* and *YTHDC1* expression were positively correlated with CD4+ T cell infiltration, while being negatively correlated with macrophage and NK cell infiltration (Figures 5A, 6A). *METTL14, ZC3H13*, and *YTHDC1* mutations were prevalent in EC (Supplementary Figures 4A, 5A, 6A). Violin chart analysis indicated that METTL14 mutations were associated with CD8+ T cell, CD4+, T cell, and B cell infiltration (Figure 4B). ZC3H13 mutations were associated with CD8+ T cell, CD4+ T cell, B cell, neutrophil, macrophage, and NK cell infiltration (Figure 5B). YTHDC1 mutations were associated with CD8+ T cell, CD4+ T cell, Treg, B cell, macrophage, and NK cell infiltration (Figure 6B). The SCNA status of *METTL14* was associated with CD8+ T cell, CD4+ T cell, Treg, macrophage, and NK cell infiltration (Figure 4C). *ZC3H13* SCNA was associated with CD8+ T cell infiltration (Figure 5C). The SCNAs of *YTHDC1* had no statistically significant relationship with immune cell infiltration (Figure 6C). Supplementary Figures 4B, 5B, 6B depict the SCNA status of *METTL14*, *ZC3H13*, and *YTHDC1* in diverse cancer types. The outcome module indicated the relevance of infiltrating immune subsets to tumor clinical stage (Figures 4D, 5D, 6D). Spearman analysis revealed that, in various cancers, the expression of METTL14, ZC3H13, and YTHDC1 was positively correlated with programmed death-ligand 1 (PD-L1) expression (Figure 7A). The same was observed for EC (Figure 7B).

### *ZC3H13* and *YTHDC1* knockdown promotes the proliferation and invasion of Ishikawa and HEC-1A cells

**Figure 8:**
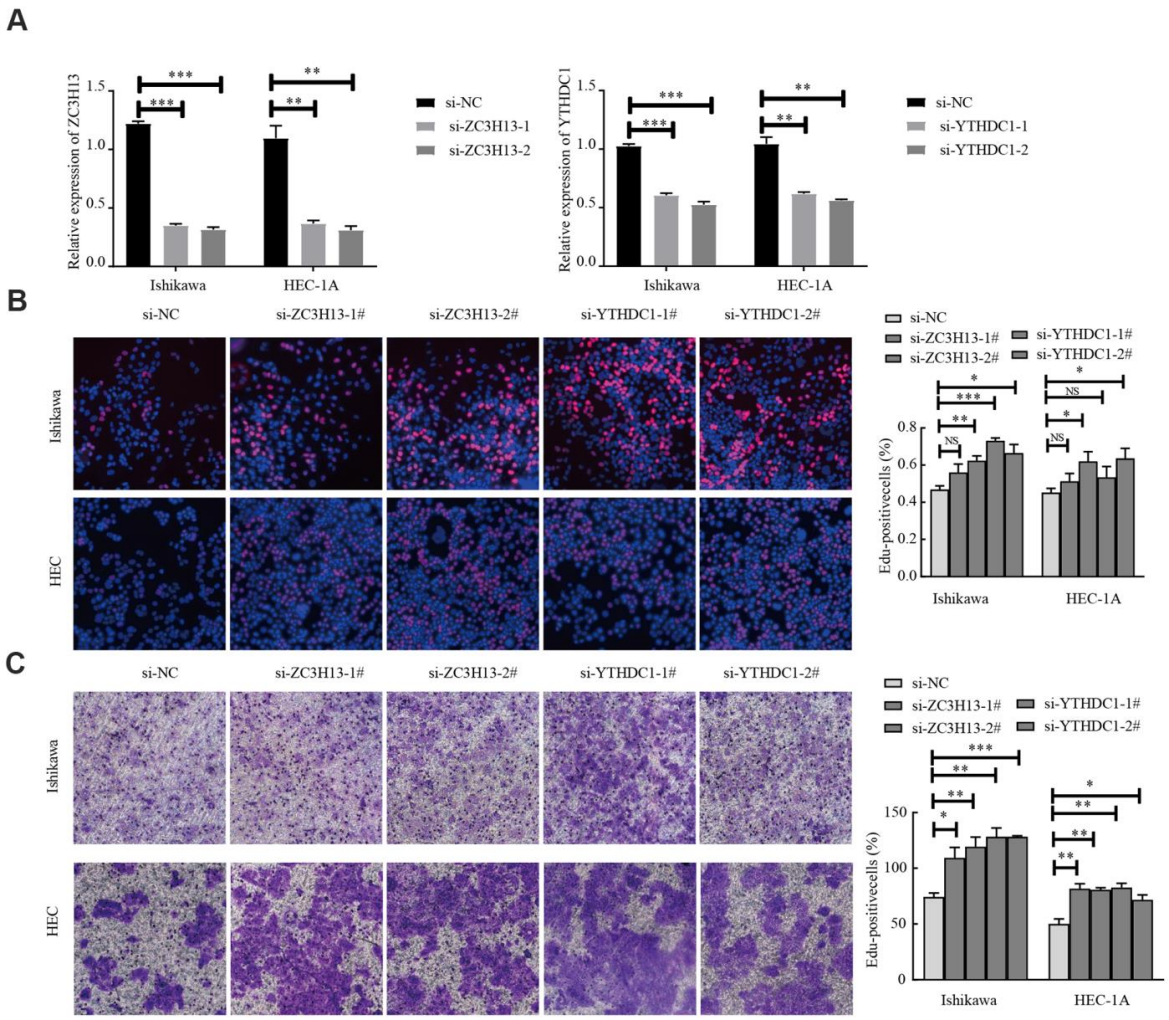
**Knockdown of ZC3H13 or YTHDC1 promotes the malignant behavior of Ishikawa and HEC-1A cells.** (**A**) qRT-PCR was used to determine transfection efficiency. (**B**) The effect of ZC3H13 or YTHDC1 expression on the proliferation of Ishikawa and HEC-1A cells was determined via EdU assays. (**C**) Transwell assays were used to determine the number of invading cells. Data are presented as the mean ± SEM (n = 3 per group), *P < 0.05, ** *P* < 0.01, *** *P* < 0.001 vs. the NC group.

EC is divided into two types, of which type I is estrogen-dependent, while type II is non-estrogen-dependent. The Ishikawa cell line is derived from type I tumors, and HEC-1A cells derived from type II tumors [21]. To investigate the carcinogenic effect of ZC3H13 and YTHDC1 in endometrial carcinoma, we knocked them down in Ishikawa and HEC-1A cell lines. The transfection efficiency of *ZC3H13*- and *YTHDC1*-targeting small interfering RNA (siRNA) was determined via quantitative RT-PCR (qRT-PCR; Figure 8A). *ZC3H13* and *YTHDC1* knockdown both promoted the proliferation of EC cell lines, as determined through EdU assays (Figure 8B). Furthermore, both si-ZC3H13- and si-YTHDC1-transfected cells exhibited stronger invasive ability compared to control cells (Figure 8C).

### Potential regulatory mechanisms of *ZC3H13* and *YTHDC1*

**Figure 9:**
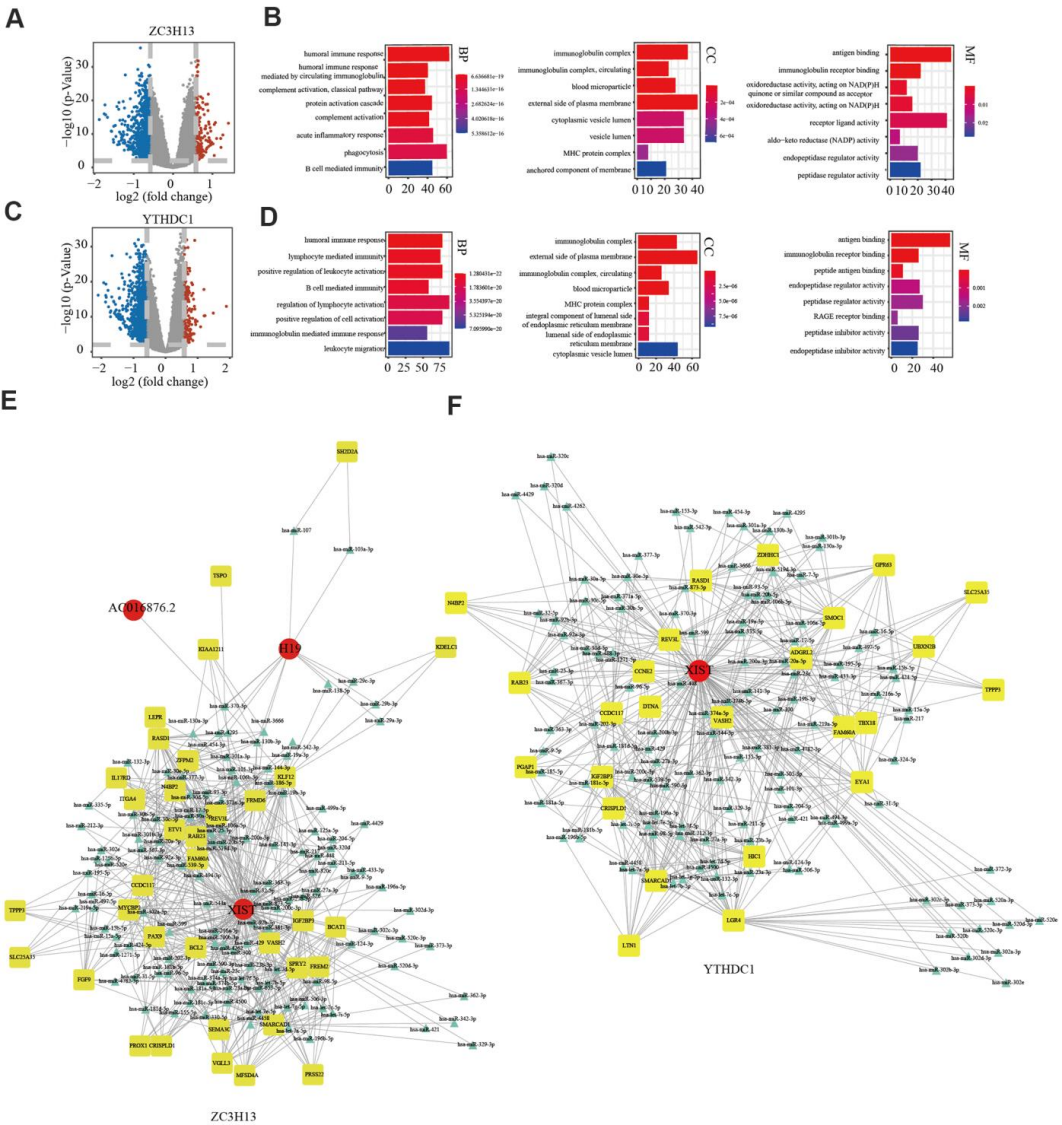
**Potential regulatory mechanism of ZC3H13 and YTHDC1 dysregulation in endometrial cancer.** (**A**) Volcano map showing the differential mRNA, microRNA, and lncRNA expression in patients with low ZC3H13 expression. An absolute log2-fold change (FC) > 1.5 and FDR-adjusted *P* < 0.01 were used as the cutoff for significantly differentially expressed mRNAs and lncRNAs. Red represents significantly upregulated genes. Blue represents genes that are significantly downregulated. Grey represents genes that are not differentially expressed. (**B**) The bar graph shows that ZC3H13-regulated expression was enriched for various GO terms. (**C**) Volcano plot showing differentially expressed transcripts in patients with low YTHDC1 expression. (**D**) The bar graph shows that YTHDC1-regulated expression was enriched for various GO terms. (**E**, **F**) The ceRNA regulatory network in patients with low expression of ZC3H13 and YTHDC1. Red indicates lncRNA; yellow indicates mRNA; blue indicates microRNA.

In view of *ZC3H13* and *YTHDC1* suppression promoting proliferation and invasion, we sought to further explore the underlying molecular mechanisms of both in EC. To this end, we downloaded lncRNA and miRNA data of 529 EC samples and 35 normal samples from the TCGA and cBioPortal for Cancer Genomics databases. A total of 846 differentially expressed genes were identified for *ZC3H13*, including 146 upregulated and 700 downregulated genes, in addition to 55 differentially expressed non-coding transcripts, 8 of which were upregulated and 47 downregulated. A total of 1107 differentially expressed genes were identified for *YTHDC1*, comprising 177 upregulated genes and 930 downregulated genes. In addition, 66 differentially expressed non-coding transcripts were identified, including 5 upregulated and 61 downregulated molecules. The bar plot depicts significantly enriched GO terms in patients with low *ZC3H13* and *YTHDC1* expression. Functional enrichment analyses revealed significantly enriched terms, including the humoral immune response and B cell-mediated immunity (Figure 9A–9D). We constructed a competing endogenous RNA (ceRNA) regulatory network to explain the potential regulatory mechanism of low *ZC3H13* and *YTHDC1* expression in EC. The ceRNA network related to *ZC3H13* expression included 3 lncRNAs, 124 miRNAs, and 35 mRNAs, while that related to *YTHDC1* expression included 1 lncRNA, 121 miRNAs, and 125 mRNAs (Figure 9E, 9F). There were 1301 differentially expressed genes associated with *METTL14*, including 186 upregulated genes and 1115 downregulated genes, in addition to 143 differentially expressed non-coding transcripts, of which 5 were upregulated and 138 were downregulated (Supplementary Figure 7A). Functional enrichment results of low *METTL14* expression in EC revealed several immune-related GO terms (Supplementary Figure 7B). The ceRNA regulatory network related to *METTL14* expression included 3 lncRNAs, 12 miRNAs, and 8 mRNAs (Supplementary Figure 7C).

### Ligand preparation and molecular docking

**Figure 10:**
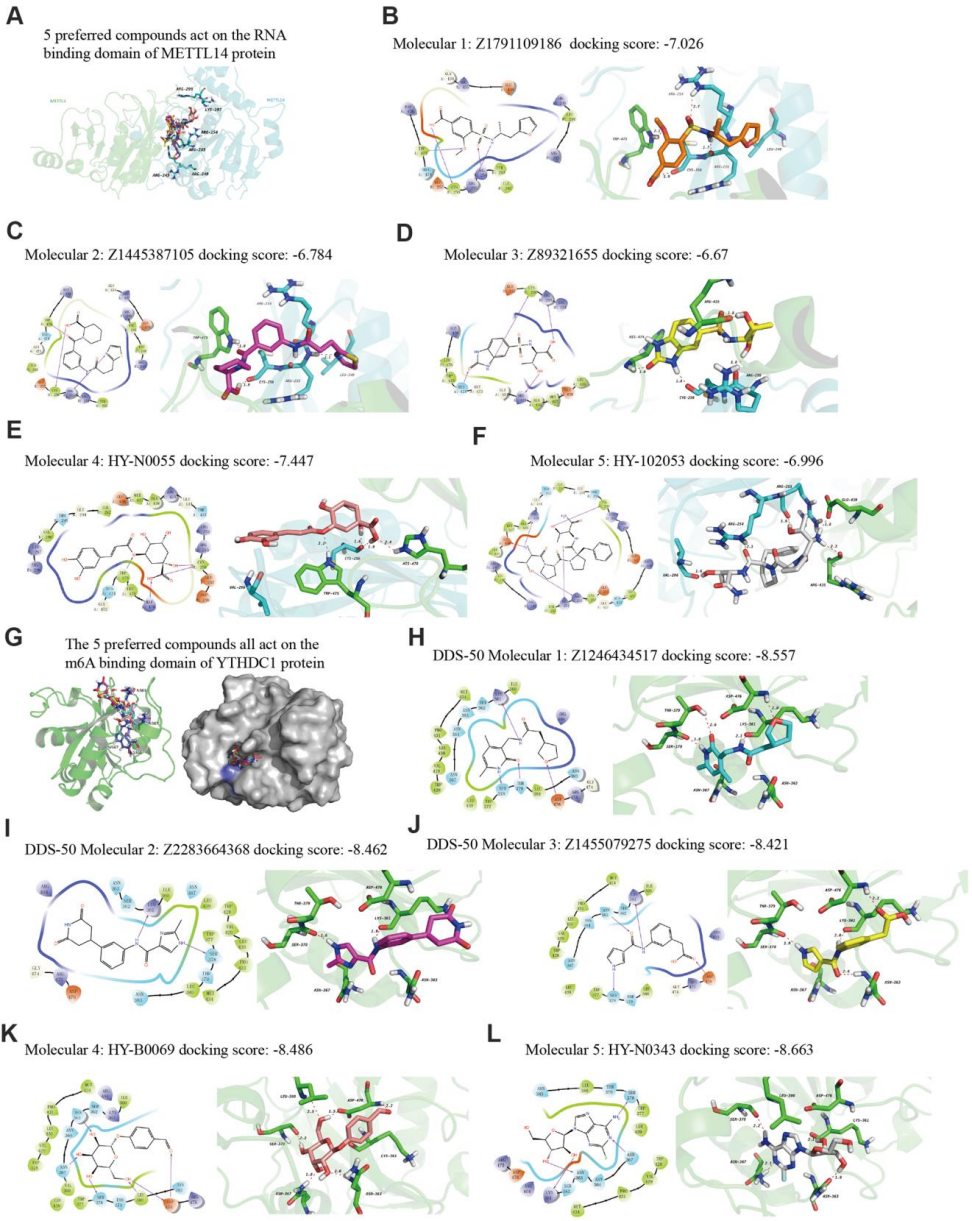
**The 2D and 3D structures of five top-scoring small-molecule drugs.** (**A**) Top five compounds acting on the RNA-binding domain of METTL14. (**B**–**F**) 2D and 3D mapping of the binding interaction between the top five compounds (Z1791109186, Z1445387105, Z89321655, HY-N0055, and HY-102053) and METTL14. (**G**) The five preferred compounds targeting the m6A-binding domain of the YTHDC1 protein. (**H**–**L**) 2D and 3D mapping of the binding between the top five compounds (Z1246434517, Z2283664368, Z1455079275, HY-N0343, and HY-B0069) and YTHDC1.

Finally, we explored drugs targeting METTL14 and YTHDC1 with potential therapeutic effects via virtual screening. In particular, we analyzed the molecular docking binding force of small-molecule compounds. The RNA-binding domain of METTL14 lies within both METTL3 and METTL14 [22]. We targeted this domain of the human METTL14 protein (key amino acids: R245/R249/R254/R255/K297/R298). Studies have reported the co-crystal structure of human YTHDC1 (PDB ID: 4R3I) and m6A, detailing the conserved m6A-binding region of YTHDC1 and the key amino acid residues (N363/N367/W377/S378) [23], which were used in the present study. From the resulting 2D and 3D model diagrams, we identified the five most relevant compounds (Figure 10).

## DISCUSSION

The initiation and progression of EC is a complex multistep process. Most of the human genome is transcribed to form a complex RNA network. However, only 1% to 2% of transcripts are translated into proteins [24, 25]. Therefore, the post-transcriptional regulation of RNA plays a key role in controlling gene expression and inevitably affects tumor cell function and cell fate [26]. Although accumulating evidence has shown that m6A regulators affect the occurrence and development of tumors [27], the role of m6A regulators in EC is poorly understood. In the current study, we analyzed CNV changes in 17 m6A regulator-encoding genes using EC TCGA data. All 17 m6A regulators harbored CNV changes. Among them, writer gene *KIAA1429* and reader gene *YTHDF3* were particularly affected by CNVs. Writer genes and reader genes play critical roles in the regulation of EC. We found that m6A regulators, with or without CNV, were related to the clinical stage, depth of invasion, and lymph node metastasis in EC patients, indicative of the contribution of m6A dysregulation to EC progression.

The treatment of EC is complicated by late-stage detection as well as recurrence and metastasis. As immunotherapy offers new therapeutic options for EC patients, an in-depth understanding of m6A regulators related to tumor-infiltrating immune cells may provide valuable insight for EC immunotherapy. By analyzing the relationship of mutations in m6A regulatory genes with *POLE, PTEN, PIK3CA, KRAS, PIK3R1, TP53, FBXW7,* and *PPP2R1A* mutations, we observed that changes in the former were significantly related to alterations in *POLE, PTEN, KRAS, TP53,* and *PPP2R1A*. Moreover, EC has heterogenous tumor morphology, clinical parameters, and genetic makeup [28]. Hence, a molecular classification system has been proposed to improve EC stratification. Many scholars have suggested new therapeutic targets through comprehensive analysis of complex molecular regulatory networks. Through survival analysis, we found that EC patients with deep or shallow deletion changes in *IGF2BP1*, *ZC3H13*, *METTL14*, *ALKBH5*, and *YTHDC1* had a poor prognosis. Further, TCGA data analysis revealed the low expression of these genes in EC tissues. We also determined their expression in normal endometrial tissues via RT-PCR. *ZC3H13, METTL14,* and *YTHDC1* expression were significantly reduced in EC tissues. Immunohistochemistry results confirmed their low expression in EC tissues at the protein level. Further, we determined that high ZC3H13, METTL14, and YTHDC1 expression was positively correlated with survival rate, yet the association was not significant. Combined with the survival curve of the EC prognostic signature from TCGA data, our results indicated that ZC3H13, METTL14, and YTHDC1 were potential m6A-related prognostic factors.

The immune system facilitates anti-tumor immune surveillance. Innate immune cells include NK cells, eosinophils, basophils, and phagocytes, which directly kill tumor cells or trigger the activation of adaptative immunity. The adaptive immune system functions through the activation and differentiation of lymphocytes (including B cells and T cells). B cells play a major role in humoral immune responses, while T cells drive cell-mediated immunity [29]. TIMER database analysis indicated that METTL14 was positively correlated with CD4+ T cell infiltration and negatively correlated with macrophage infiltration. ZC3H13 and YTHDC1 were also positively correlated with CD4+ T cell infiltration while being negatively correlated with macrophage and NK cell infiltration. *METTL14*, *ZC3H13*, and *YTHDC1* mutations were frequently detected in EC. *METTL14* mutations were associated with CD8+ T cell, CD4+, T cell, and B cell infiltration. *ZC3H13* mutations were associated with CD8+ T cell, CD4+ T cell, B cell, neutrophil, macrophage, and NK cell infiltration. Mutations of *YTHDC1* were associated with CD8+ T cell, CD4+ T cell, Treg, B cell, macrophage, and NK cell infiltration. Taken together, TIMER analysis indicated that METTL14, ZC3H13, and YTHDC1, as key regulators of m6A modifications, were closely related to tumor immune infiltration in EC.

Cancer cells have evolved a variety of mechanisms for immune evasion, including defective antigen presentation, upregulation of immune checkpoint molecules, and the recruitment of immunosuppressive cell populations, all of which contribute to the failure of anti-tumor immune responses [30]. As a key immune checkpoint, PD-L1 inhibits anti-tumor immunity by interacting with its receptor, programmed cell death protein-1 (PD-1) on the membrane of immune cells. Blocking the PD-L1/PD-1 interaction can significantly enhance the anti-tumor immune response and represents a major breakthrough in cancer treatment [31]. In recent years, data from clinical studies on immune checkpoint inhibition in EC have demonstrated the efficacy of PD-1 inhibitors in a subgroup of advanced PD-L1-positive endometrial cancer patients who failed previous multi-line treatments [32]. However, studies have shown that immunotherapy-resistant colorectal cancer (CRC) tumors have a low mutation burden with proficient mismatch repair or low microsatellite instability (pMMR-MSI-L). The inhibition of METTL3 and METTL14 via methyltransferase inhibitors suppressed N-methyladenosine (mA) mRNA modification and enhanced the response to anti-PD-1 therapy in pMMR-MSI-L CRC and melanoma, highlighting the relevance of RNA methylation in adaptive immunity [33]. Our analysis revealed that the expression of METTL14, ZC3H13, and YTHDC1 was positively correlated with PD-L1 expression in EC, suggesting that these proteins can improve the immunotherapy outcome.

ZC3H13 is a classical CCCH zinc finger protein, whose gene is located on human chromosome 13q14.13 [34]. Studies have shown that following ZC3H13 knockdown, the level of m6A mRNA is reduced, with ZC3H13 playing a key role in the regulation of RNA m6A methylation within the nucleus [35]. In CRC, ZC3H13 downregulates the expression of Snail, Cyclin D1, and Cyclin E1 by inhibiting Ras-ERK signaling, in turn also suppressing proliferation and invasion [36]. However, ZC3H13 expression and its cellular mechanism in EC remain poorly understood. By interfering with the expression of ZC3H13 in Ishikawa and HEC-1A cells, we observed that ZC3H13 promotes the proliferation and invasion of EC cells. To further explore the molecular mechanism of ZC3H13 in EC, a ceRNA regulatory network was constructed, including 3 lncRNAs, 124 miRNAs, and 35 mRNAs. Among these, lncRNA H19 was determined to have a key molecular role within the network. Studies have shown that H19 is highly expressed in EC tissues, and overexpression of lncRNAH19 promoted proliferation by upregulating Snail in EC cells [37].

YTHDC1, as a reader of the m6A methylation, can recognize and bind m6A-modified sites within RNA. A previous study revealed that YTHDC1 protein expression was reduced in EC tissues, and patients with high YTHDC1 expression had higher OS and DFS [38], which is consistent with our results. However, the exact underlying mechanism of YTHDC1 in EC cells remains poorly understood. Knockdown of YTHDC1 in Ishikawa and HEC-1A cells promoted their proliferation and invasion. Further, miR-200c was an important molecule within the YTHDC1 ceRNA regulatory network. Studies have shown that miR-200c regulates the malignant phenotype of HEC-1A and Ishikawa cells by modulating the nuclear translocation of β-catenin [39].

The lncRNA X-inactive specific transcript (XIST) mediates the transcriptional silencing of genes on the X chromosome. XIST is hypermethylated in human cells, having at least 78 m6A sites [40]. Moreover, the XIST lncRNA is a downstream target of METTL14. Silencing METTL14 essentially eliminates the m6A level in XIST, enhancing its expression as well as the proliferation and invasion ability of CRC cells *in vitro* [41]. The YTHDC1 reader protein preferentially recognizes XIST [42]. We constructed the ceRNA regulatory network of YTHDC1 to explore new directions for studying its molecular mechanism. Approximately 70% of tumor samples in EC were previously reported to exhibit reduced m6A levels, primarily due to a loss-of-function METTL14 mutation at the R298P site and activation of the AKT pathway to enhance EC proliferation and tumorigenesis [43]. We found that the expression of METTL14 was reduced in EC, and miR-206 was a key molecule in the ceRNA network of METTL14. Previous studies have shown that miR-206 targets HDAC6 to regulate EC progression through the PTEN/AKT/mTOR pathway [44]. Further, molecular ALKBH5 inhibitors enhanced the efficacy of immunotherapy for melanoma, CRC, and other cancers [45]. To explore small-molecule compounds as potential therapeutics, we downloaded the protein crystal structures of human METTL14 and YTHDC1 from RCSB PDB. There is no experimentally-determined three-dimensional structure for ZC3H13 or an appropriate homologous structure. The docking data generated herein may be of use for the synthesis of new drug candidates and the identification of novel drug targets for molecular therapy. Further, future studies using m6A-seq and m6A-modified RNA immunoprecipitation (MeRIP) will contribute to a more in-depth analysis of m6A regulatory factors in EC.

In summary, we analyzed TCGA EC patient data, including sequence and CNV data, determining the genetic alterations of m6A regulatory genes in EC. We observed a clear relationship between alterations resulting in decreased m6A levels and poor clinical outcomes. We identified ZC3H13, METTL14, and YTHDC1 as independent prognostic factors in EC patients. Notably, our results show that the expression, mutation, and SCNA of *METTL14, ZC3H13*, and *YTHDC1* were related to immune cell infiltration. Further, the expression of these three genes was positively correlated with PD-L1 expression in EC. Meanwhile, *in vitro* knockdown of *ZC3H13* or *YTHDC1* promoted the malignant phenotype of EC cells. Taken together, we provided new molecular prognostic markers for EC as well as potential therapeutic targets through the construction of ceRNA regulatory networks, while also identifying potentially effective EC drugs.

## MATERIALS AND METHODS

### Data sources and processing

We obtained relevant clinical, somatic mutation, CNV, and RNA-seq data from the UCSC Xena database (https://xenabrowser.net/datapages/). Protein expression in EC tissues was analyzed using the UALCAN (http://ualcan.path.uab.edu/index.html) online tool. The GISTIC algorithm was used to examine CNV and assigned estimated values to -2, -1, 0, 1, and 2, representing homozygous deletion, single copy deletion, diploid normal copy, low-level copy number amplification, or high-level copy number amplification. To investigate the clinicopathological significance of CNV and mutations, we divided TCGA EC cohort data into two groups: with mutation and/or CNV and without CNV or mutation. The mRNA expression data were calculated from RNA-Seq V2 RSEM, applying log scale before analyzing the relationship between mRNA expression and CNV. Scores for six types of immune cells (B cell, CD4 T cell, CD8 T cell, Treg, neutrophil, macrophage, and natural killer (NK) cell) were obtained based on mRNA expression data using the TIMER package (https://cistrome.shinyapps.io/timer/).

### Differential expression analyses and gene set enrichment analysis

We downloaded EC RNA-seq, miRNA-seq, and clinical data from TCGA database. According to the Ensembl (Homo sapiens; https://asia.ensembl.org/index.html) database, gene IDs were converted into gene names. miRNA-seq data were analyzed via the same method. Differentially expressed lncRNAs, mRNAs, and miRNAs were screened using the edgeR program package in R software. Differentially expressed transcripts had an absolute fold change (FC) >1.5 and *P* < 0.01. We used the R program to construct a volcano map of differentially expressed genes (DEGs). An absolute log2-FC > 1.5 and false discovery rate (FDR)-adjusted *P* value < 0.01 were used as the cutoff for significantly differentially expressed mRNAs (DEMs) and lncRNAs (DELs).

### Construction of the ceRNA regulatory network

GO and KEGG enrichment analyses were carried out using the clusterProfiler R package. A Benjamini and Hochberg-adjusted *P* < 0.05 was considered statistically significant. DEGs were compared in public expression databases miRcode, miRTar Base, TargetScan, and miRDB. The lncRNA-miRNA-mRNA interaction was divided into respective lncRNA-miRNA and miRNA-mRNA interactions. By comparing lncRNA and miRNA with the miRcode database, predictions were made regarding lncRNA-miRNA interactions. We then cross-referenced the prediction results with the miRTarBase, TargetScan, and miRDB databases in order to obtain differentially expressed miRNA regulatory genes. Cytoscape v3.5.117 was used to visualize and map the network.

### Patients and samples

We selected patients who had undergone surgical removal of the uterus at the Department of Gynecology, Shengjing Hospital, affiliated to China Medical University, from 2011 to 2017. We collected 34 samples of EC tissue and 20 samples of normal endometrial fresh tissue. In addition, 40 paraffin-embedded specimens, including 35 EC tissues and 20 normal endometrial tissues, were obtained. No patient had received anti-cancer treatments, including radiotherapy, chemotherapy, or immunotherapy, before surgery. The age range was 23–69 years, and histopathological analysis was performed by two pathologists. This study was approved by the Ethics Committee of Shengjing Hospital, affiliated to China Medical University (ethical approval number: 2018PS251K).

### Cell culture

Ishikawa cells were cultured in RPMI 1640 medium (Gibco, Carlsbad, CA, USA), while HEC-1A cells were cultured in McCoy’s 5A medium (Gibco). Both cell lines were obtained from the Institute of Biochemistry and Cell Biology, Chinese Academy of Sciences (Shanghai, China).

### Transfection of Ishikawa and HEC-1A cells

Cells were transfected with siRNAs against ZC3H13 (si-ZC3H13) and YTHDC1 (si-YTHDC1) (GenePharma, Shanghai, China) using Lipofectamine™ 3000 (ThermoFischer, Carlsbad, CA, USA) as per the manufacturer instructions. si-NC (GenePharma, Shanghai, China) was used to transfect control cells. The si-RNA sequences are listed in Supplementary Table 1.

### Cell proliferation assay

Ishikawa and HEC-1A cells at the logarithmic growth phase were uniformly suspended and transferred to 96-well plates. A blank control with three parallel wells was included in each group. An EdU cell proliferation detection kit (RiboBio, Guangzhou, China) was used to assess the cell proliferation capacity as per the manufacturer’s instructions. Briefly, 50 μM of EdU mixed reagent was prepared, and the cells were incubated with it for 2 h. After the cells were fixed, DNA staining was performed, and cells were washed with PBS. Image acquisition and analysis were performed using a fluorescence microscope (Nikon, Japan) at 20× the original magnification.

### Cell invasion assay

After the Transwell chamber was sterilized, Matrigel (pore size 8 μm; Corning, NY, USA) was added and incubated at 37° C overnight for the gel to solidify. Cells in the logarithmic growth phase were harvested, centrifuged, and the supernatant was discarded. The cells were resuspended in serum-free medium. Subsequently, 800 μL of culture medium (containing 10% fetal bovine serum) was added to another 24-well plate, and the Transwell chamber was transferred into the 24-well plate. The cell suspension (5 × 104 cells) was added to the upper chamber and incubated for 24 h at 5% CO2 and 37° C. The Transwell chamber was then removed. The non-adherent cells in each well were removed, and the upper chamber liquid was discarded. After the well was air-dried, it was fixed in 4% paraformaldehyde. Crystal violet dye solution was added, and cells were rinsed with PBS. Images were acquired under a microscope, and cells were counted.

### RNA extraction and quantitative RT-PCR

TRIzol reagent (Takara, Shiga, Japan) was used to extract total RNA from cultured cells and tissues. Reverse transcription of total RNA into cDNA was performed with PrimeScript™ RT-PCR Kit (Takara) and SYBR® TB Green™ Premix Ex Taq II (Takara) for real-time PCR analysis. Specific PCR primers were designed by Sangon Biotech Co., Ltd. (Shanghai, China). The fold change in expression was calculated using the 2^-∆∆Ct^ method, with *GAPDH* as an internal control. The primer sequences are listed in Supplementary Table 2.

### Immunohistochemistry

Immunohistochemistry was carried out to detect the protein expression of ZC3H13, YTHDC1, and METTL14. The primary antibodies used are listed in Supplementary Table 3. Staining was primarily localized within the nucleus, seen as brown foci. The scoring method for positive expression was based on whether the cytoplasm had a brownish yellow or brown color. Samples were scored as follows: no staining, 0; light yellow, 1; yellow, 2; brown or sepia, 3. The scores were assigned according to the percentage of positive cells, with a negative count denoted as 0. The percentage of positive cells was scored as follows: < 10 % = 1, ≥ 10–50 % = 2, > 50–75 % = 3, and ≥ 75 % = 4. The product of the two scores was considered as the total score, and the results were interpreted as follows: ≤ 2 = negative, 3–4 = weak positive (+), 5–8 = medium positive (++), and 9–12 = strong positive (+++). Low expression was indicated by -/+, and high expression was indicated by ++/+++. The results were evaluated by two senior pathologists who were blinded to the patients’ data. Each sample was independently observed to determine the positive cell count and evaluate the background. In cases of disagreement, a third pathologist made the judgement.

### Virtual screening process

The crystal structure of the target protein was obtained from the protein database (PDB database, https://www.rcsb.org/). Next, we used the Protein Preparation Wizard module to hydrogenate the protein, remove water molecules, SAM, ethylene glycol, and SEP, repair missing residues, and add side chains. The LigPrep Module was used to perform hydrogenation, energy optimization, and to construct the 3D structure. The Virtual Screening Workflow module was used for virtual screening. After importing the prepared compound, the Glide module was used for molecular docking, that is, the receptor and ligand molecules were aligned with each other through geometric and energy matching. The higher the absolute value of the docking score, the stronger the binding force.

### Statistical analysis

We used GraphPad Prism 8 software (GraphPad, Inc., La Jolla, CA, USA) for statistical analysis. The difference in count data was assessed via the χ^2^ test, and data are expressed as mean ± SEM. Wilcoxon’s signed-rank test was used to compare the immune cell infiltration between mutant and wild-type expression data. The difference between different CNV types were assessed using the Kruskal-Wallis test. Kaplan-Meier analysis and the log-rank test were used to compare the OS and DFS between patients with different CNV types. DFS was defined as the time from the date of diagnosis to the time of progression/death or last follow-up. All statistical tests were two-sided and implemented in the R programing language. *P* < 0.05 was considered statistically significant.

### Ethics approval and consent to participate

The study protocol was reviewed and approved by the Scientific Research and New Technology Ethical Committee of the Shengjing Hospital of China Medical University. Ethical number: 2018PS251K.

### Consent for publication

We have obtained consent to publish this paper from all the participants of this study.

### Availability of data and materials

The datasets used during the current study are available from the corresponding author on reasonable request.

## Supplementary Material

Supplementary Figures

Supplementary Tables 1, 2 and 3

Supplementary Table 4
