# Supplementary material for: Immune infiltration-related N6-methyladenosine RNA methylation regulators influence the malignancy and prognosis of endometrial cancer
Source: Aging (Albany NY). 2021 Jun 16;13(12):16287–315. doi: 10.18632/aging.203157 (PMC8266343; doi:10.18632/aging.203157)
Supplement: Supplementary Figures [file aging-13-203157-s001.pdf]

SUPPLEMENTARY FIGURES

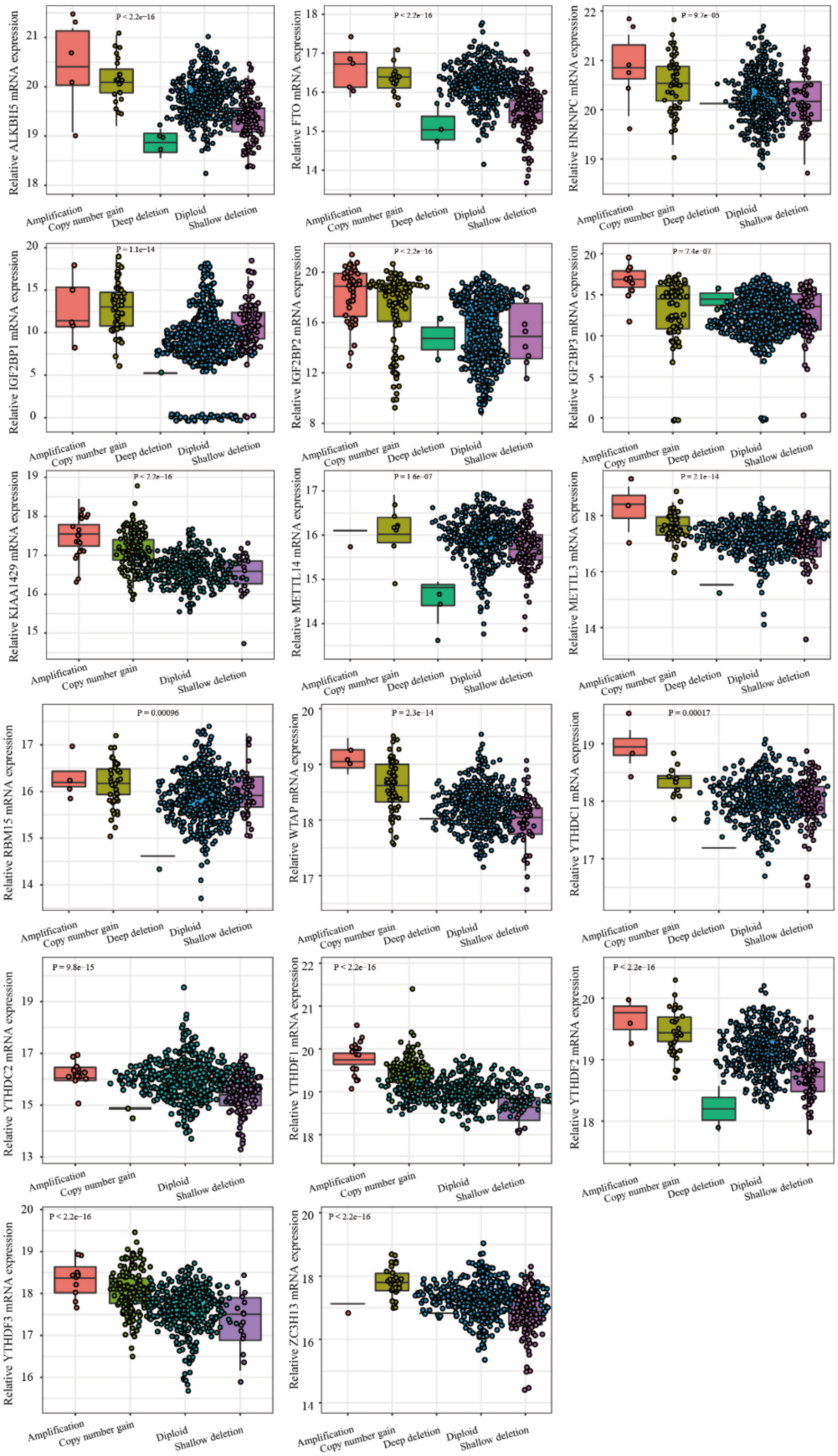

Supplementary Figure 1. Correlation between different CNV patterns of the 17 m6A regulatory genes and respective mRNA expression levels.

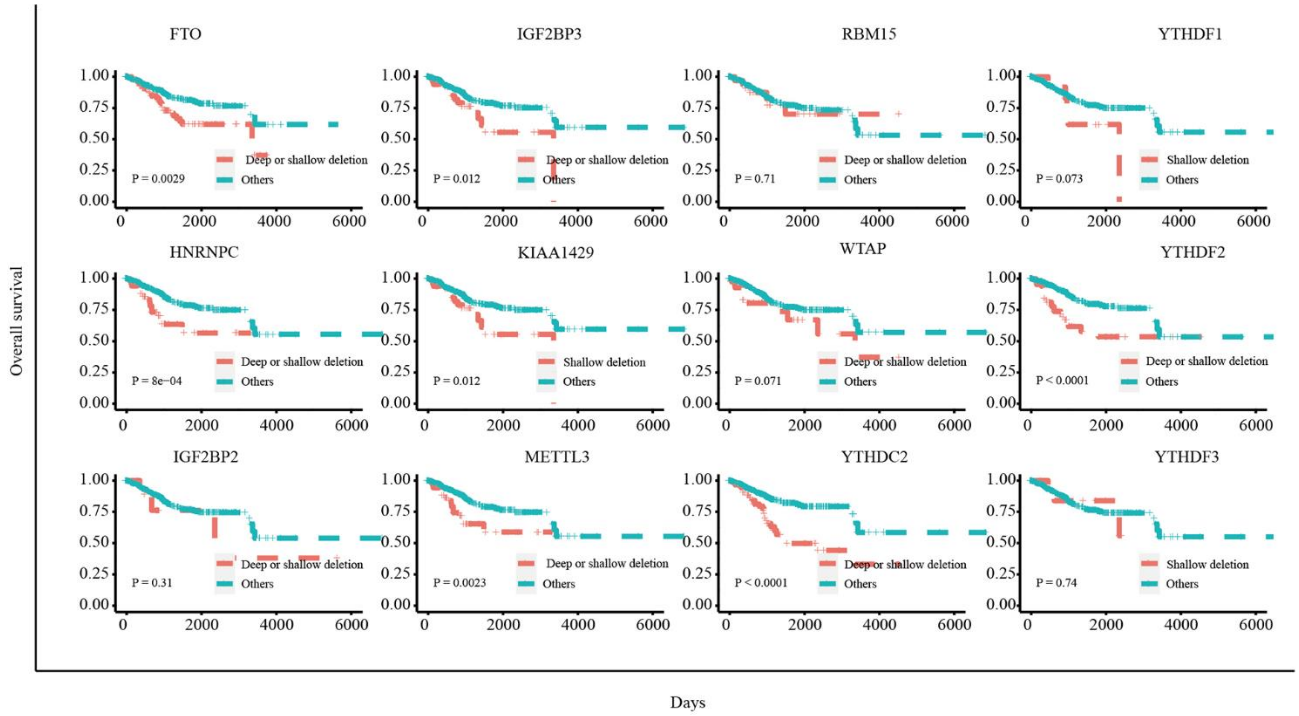

Supplementary Figure 2. The relationship between CNV of m6A regulatory genes and OS in EC patients.

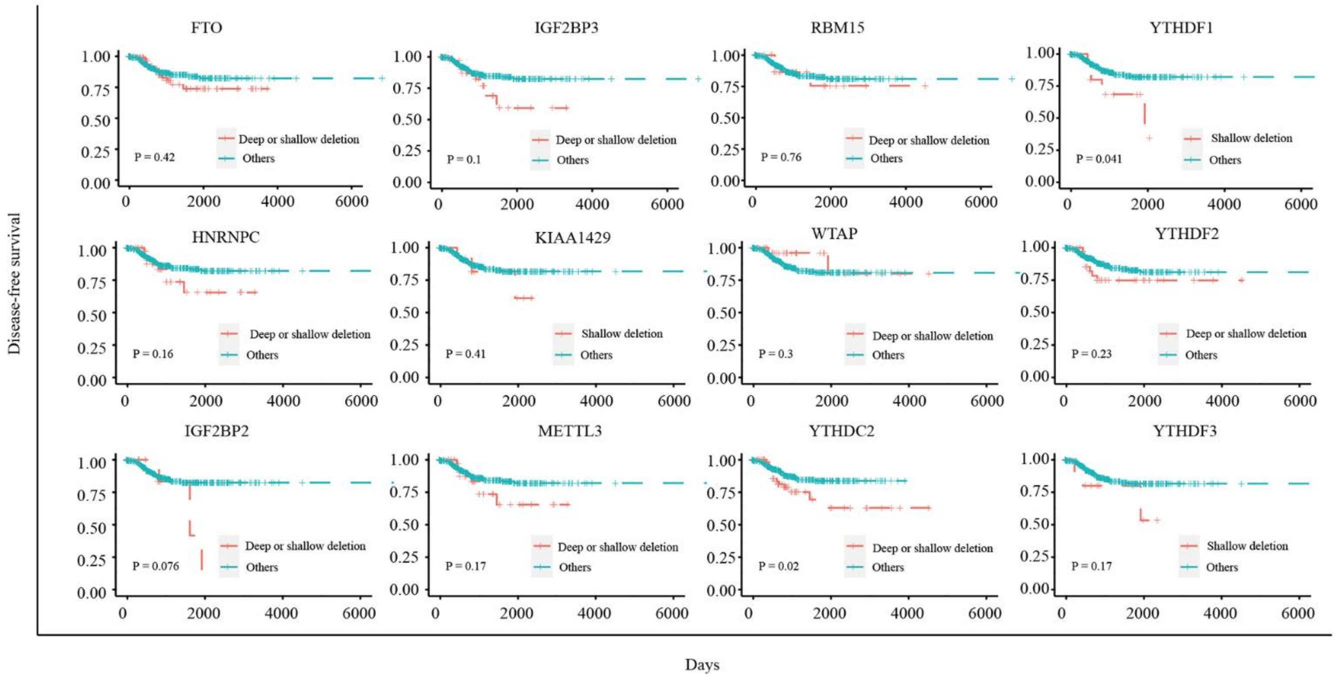

Supplementary Figure 3. The relationship between CNV of m6A regulatory genes and DFS in EC patients.

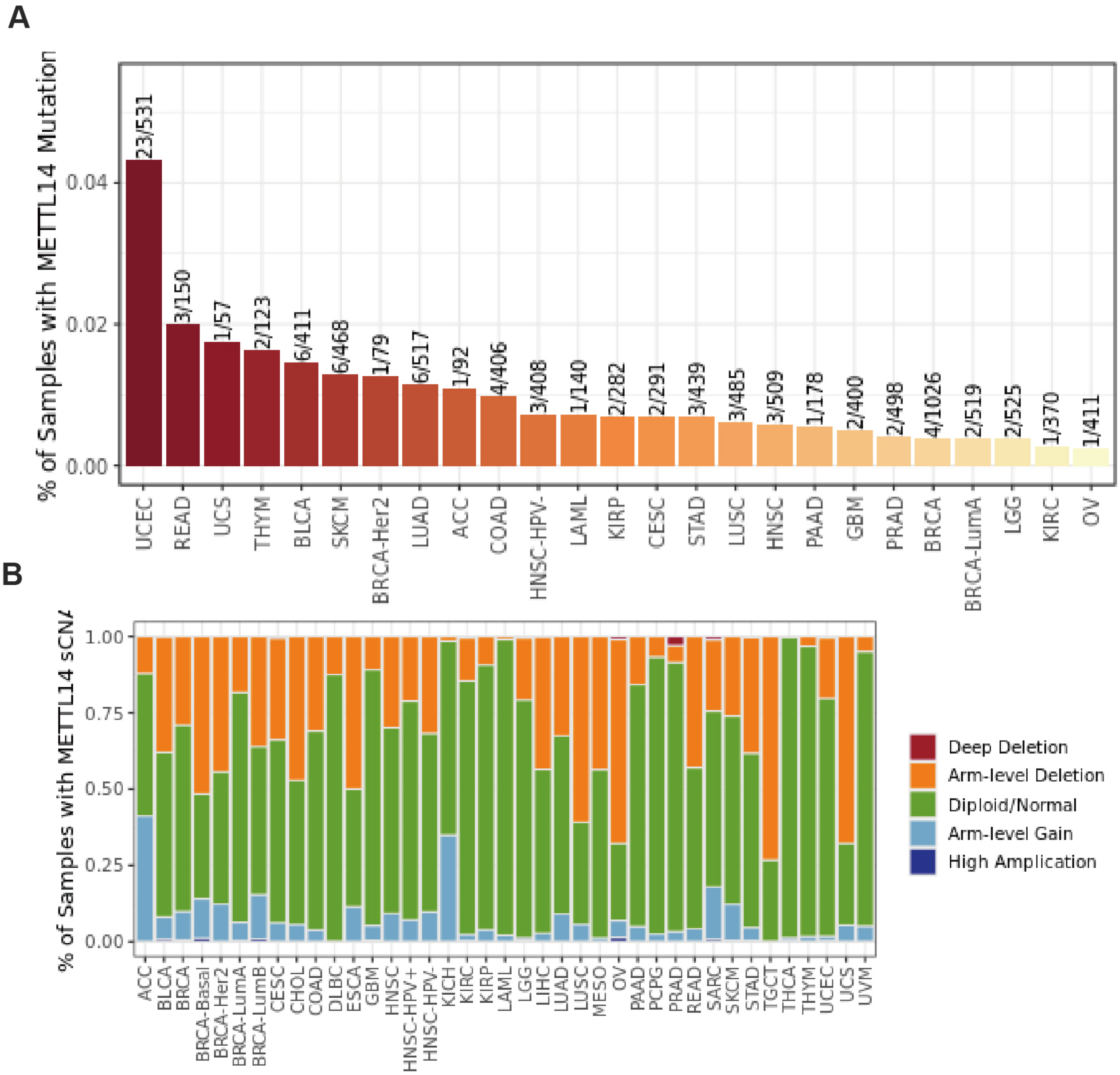

Supplementary Figure 4. METTL14 mutation, SCNA status in diverse cancer types. (**A**) Mutation status. (**B**) sCNA status.

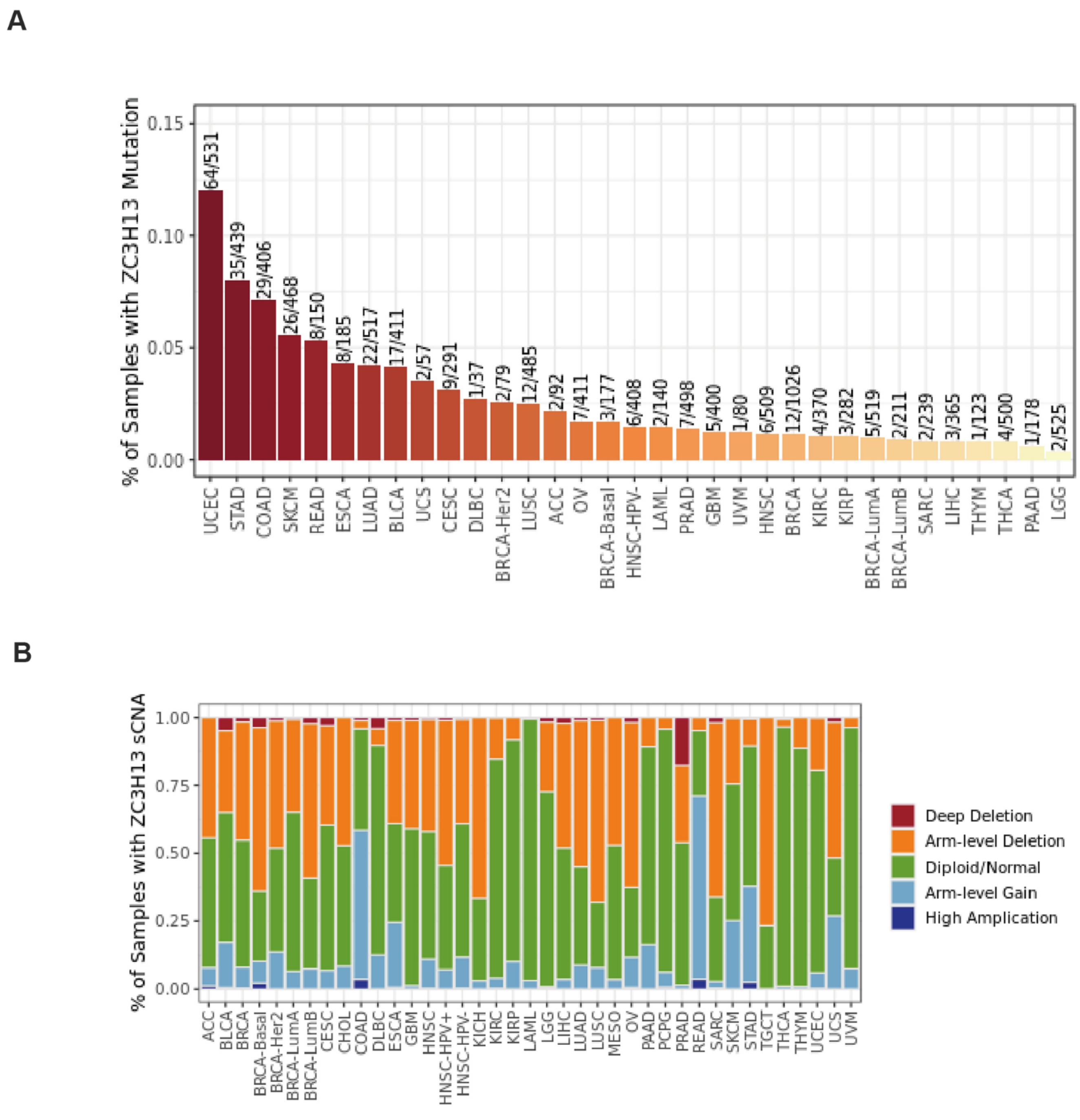

Supplementary Figure 5. ZC3H13 mutation, SCNA status in diverse cancer types. (**A**) Mutation status. (**B**) sCNA status.

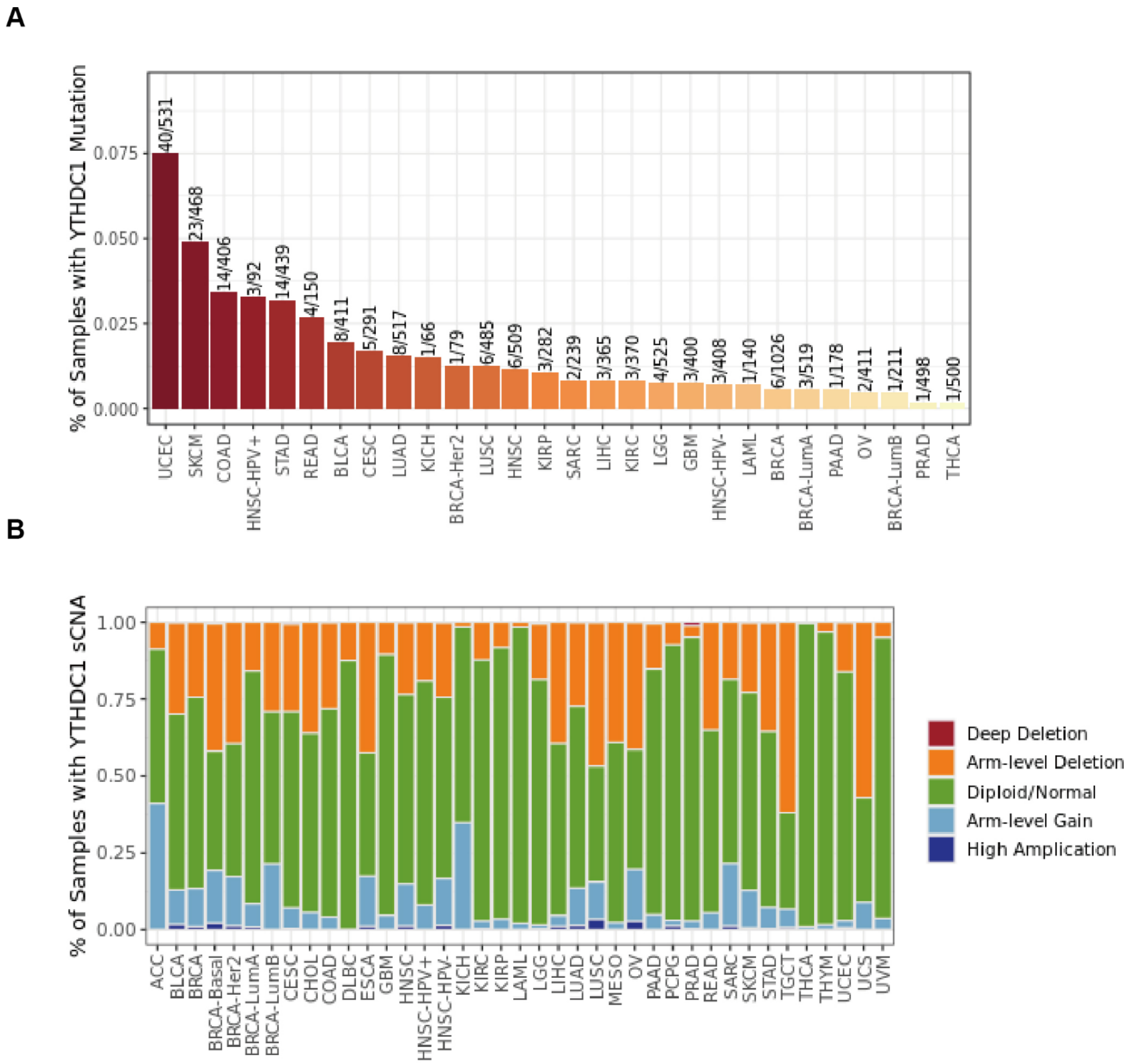

Supplementary Figure 6. YTHDC1 mutation, SCNA status in diverse cancer types. (**A**) Mutation status. (**B**) sCNA status.

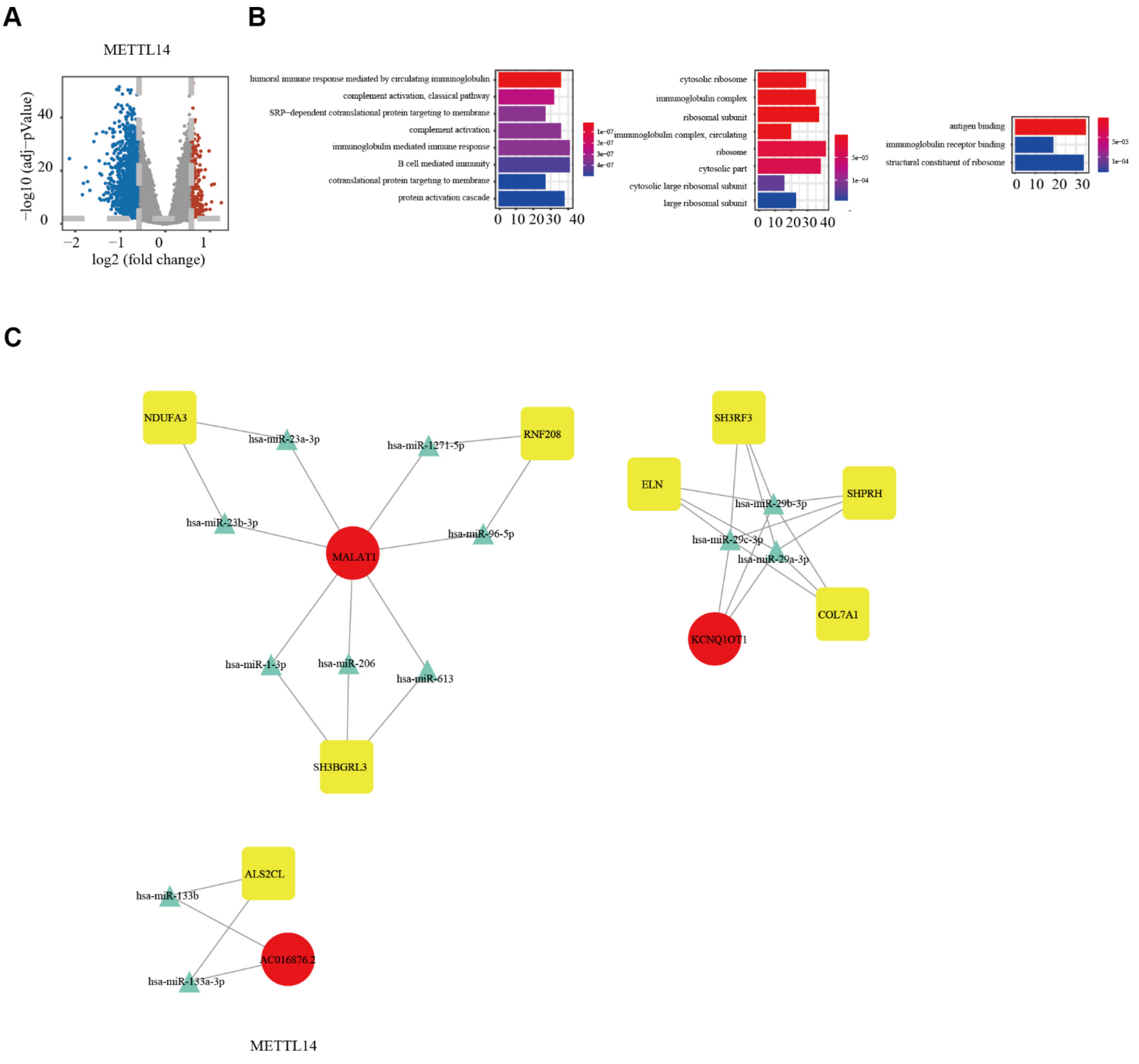

Supplementary Figure 7. Potential regulatory mechanism of METTL14 dysregulation in EC. (**A**) A volcano map shows the differential molecule, microRNA and lncRNA changes in patients with low METTL14 expression, An absolute log2-fold change (FC) > 1.5 and false discovery rate (FDR) adjusted P value < 0.01 were used as the cutoff for significantly differentially expressed mRNA (DEMs) and 1 ncRNAs (DELs). Red represents significantly up-regulated genes. Blue represents genes that are significantly downregulated. Gray represents genes that are not differentially expressed. (**B**) The bar graph shows that METTL14 is significantly rich in gene ontology terms. (**C**) The ceRNA regulatory network (ceRNA) regulatory network in patients with low expression of METTL14. Red indicates disorder of lncRNAs; yellow indicates disorder of mRNA; blue indicates disorder of microRNA.
