# Supplementary material for: Immune infiltration-related N6-methyladenosine RNA methylation regulators influence the malignancy and prognosis of endometrial cancer
Source: Aging (Albany NY). 2021 Jun 16;13(12):16287–315. doi: 10.18632/aging.203157 (PMC8266343; doi:10.18632/aging.203157)
Supplement: Supplementary Tables 1, 2 and 3 [file aging-13-203157-s002.pdf]

SUPPLEMENTARY TABLES

Supplementary Table 1. Sequences of si-RNAs used.

Name	Sequence
si-ZC3H13-1	Sense: 5'-CCGAAGACCCAGUGUAUUUTT-3'<br>Antisense: 5'-AAAUACACUGGGUCUUCGGTT-3'
si-ZC3H13-2	Sense: 5'- GGAGUCAUCCUCACCUGUUTT-3'<br>Antisense: 5'-AACAGGUGAGGAUGACUCCTT-3'
si-YTHDC1-1	Sense: 5'- GGAGAAAGAUGGAGAACUUTT-3'<br>Antisense: 5'- AAGUUCUCCAUCUUUCUCCTT-3'
si-YTHDC1-2	Sense: 5'-GCUCUGCAUCAGAGUCAUATT-3'<br>Antisense: 5'-UAUGACUCUGAUGCAGAGCTT-3'
si-NC	Sense: 5'-UUCUC GAACGUGUCACGUTT -3'<br>Antisense: 5'-ACGUGACACGUUCGGAGAATT -3'

Supplementary Table 2. PCR primer sequences.

Name	Sequence
IGF2BP1	F: AGGCAGGCTGACGAGGTTCC<br>R: GGTTCCGTCCTTCCTTGCCAATG
ZC3H13	F: CGGACAGTGATGCCTACAACAGTG<br>R: TGAGGTGCGAGGGACTAAGAGAAC
METTL14	F: ACCAAAATCGCCTCCTCCCAAATC<br>R: AGCCACCTCTTTCTCCTCGGAAG
ALKBH5	F: GCAAGGTGAAGAGCGGCATCC<br>R: GTCCACCGTGTGCTCGTTGTAC
YTHDC1	F: ATCATCTTCCGTTCGTGCTGTCC<br>R: ATACACCCTTCGCTTTGGCAAGAG
GAPDH	F: GCACCGTCAAGGCTGAGAAC<br>R: TGGTGAAGACGCCAGTGGA'

Supplementary Table 3. The primary antibodies used for immunohistochemistry.

Name	Manufacturer	Dilution ratio: western blotting, immunohistochemistry
ZC3H13	Affinity Biosciences.OH.USA	1:200
YTHDC1	Affinity Biosciences.OH.USA	1:200
METTL14	Affinity Biosciences.OH.USA	1:200
