# Supplementary material for: Immune infiltration-related N6-methyladenosine RNA methylation regulators influence the malignancy and prognosis of endometrial cancer
Source: Aging (Albany NY). 2021 Jun 16;13(12):16287–315. doi: 10.18632/aging.203157 (PMC8266343; doi:10.18632/aging.203157)
Supplement: Supplementary Table 4 [file aging-13-203157-s003.docx]

**Supplementary Table 4. Mutations of m6A regulatory genes in 433 endometrial cancer patients.**

| **UCEC sampleID** | **ALKBH5** | **FTO** | **HNRNPC** | **IGF2BP1** | **IGF2BP2** | **IGF2BP3** | **KIAA1429** | **METTL14** |
| --- | --- | --- | --- | --- | --- | --- | --- | --- |
| TCGA-AP-A0LM | NA | NA | NA | p.R452C | p.A370T | p.A209V | p.D1546Y p.E394* p.I449I p.L388L | p.K76N |
| TCGA-BK-A6W3 | NA | NA | NA | NA | NA | NA | NA | NA |
| TCGA-AX-A2HA | NA | NA | NA | NA | NA | NA | NA | NA |
| TCGA-EO-A22X | NA | p.E369* | NA | p.K272N | NA | NA | p.K1078T p.S842Y | NA |
| TCGA-AP-A1E0 | NA | NA | NA | p.G541R | NA | NA | NA | NA |
| TCGA-E6-A1LX | NA | p.E190* p.E263K | NA | p.D156N | p.F456F | NA | p.E1084E p.E926G p.K576K p.R269Q | p.R228Q |
| TCGA-A5-A2K7 | NA | NA | NA | p.D156N | NA | NA | NA | NA |
| TCGA-EO-A3B0 | NA | NA | NA | NA | NA | NA | NA | NA |
| TCGA-A5-A1OF | NA | NA | NA | NA | p.A121T | NA | p.E263E p.S861Y | p.R456* |
| TCGA-AJ-A3EL | NA | NA | NA | NA | p.I558I | p.F41C | p.Y1608* | NA |
| TCGA-AX-A0J0 | NA | p.K391Q | NA | p.A256T | p.F501L | p.I470L | NA | p.L190I |
| TCGA-AX-A1C5 | NA | NA | NA | NA | NA | NA | NA | NA |
| TCGA-B5-A1MX | p.P128S | p.C326C | p.A290D | NA | p.Q414* | NA | p.G1794* | p.R142R |
| TCGA-BG-A222 | NA | NA | NA | NA | NA | NA | p.F721Lfs*11 | NA |
| TCGA-B5-A0JY | NA | p.D348N p.E375K p.L51I p.R459Q | NA | p.N532N p.R452C p.S438S p.T56T | NA | p.L247F p.T326N | p.E1084D | NA |
| TCGA-BG-A0M8 | NA | NA | NA | NA | NA | NA | p.D750Y | NA |
| TCGA-D1-A163 | NA | NA | NA | NA | NA | NA | NA | NA |
| TCGA-D1-A16N | NA | NA | NA | NA | NA | NA | NA | NA |
| TCGA-D1-A174 | NA | NA | NA | NA | NA | NA | NA | NA |
| TCGA-EO-A3AV | NA | NA | NA | NA | NA | NA | NA | NA |
| TCGA-EY-A1GD | NA | NA | NA | p.C257C p.F33L | NA | NA | NA | NA |
| TCGA-AX-A1C4 | NA | NA | NA | NA | NA | p.I302Lfs*17 | NA | NA |
| TCGA-BG-A0MK | NA | NA | NA | NA | NA | NA | NA | NA |
| TCGA-EY-A1GI | NA | NA | NA | NA | p.V122F | NA | NA | NA |
| TCGA-AJ-A3EK | NA | NA | p.E252* | p.V521M | p.D157N | p.A283V | p.R1808H p.T728I | NA |
| TCGA-AP-A1DK | p.R324Q | p.A437V p.S358S | NA | p.I196T | p.H282H p.R597C | p.A216T p.A435T | p.S1082S | p.P147S |
| TCGA-AX-A2HC | NA | NA | NA | p.E334K p.K300E | NA | NA | p.A444V p.E527* p.I1638S p.R1163C p.S496P | NA |
| TCGA-B5-A11E | NA | NA | p.K170N | NA | p.P195P | NA | p.G1588* p.P649H | p.A279V |
| TCGA-B5-A3FC | p.A333V | p.R337H | NA | p.A382T | p.S379S | p.R290H p.R86* | NA | p.R425C |
| TCGA-BG-A18B | NA | NA | NA | p.Q409H | NA | NA | NA | NA |
| TCGA-E6-A2P8 | NA | NA | NA | NA | NA | NA | NA | NA |
| TCGA-AJ-A3OJ | NA | NA | NA | NA | NA | NA | p.F721Lfs*11 | NA |
| TCGA-D1-A16X | NA | NA | NA | NA | NA | NA | NA | NA |
| TCGA-D1-A17Q | NA | NA | p.R167Q | p.R219H | NA | p.K346T | NA | NA |
| TCGA-B5-A1MW | NA | NA | NA | NA | NA | NA | p.K1465K | NA |
| TCGA-BS-A0U8 | NA | NA | NA | NA | NA | NA | NA | NA |
| TCGA-BS-A0UV | NA | p.S482L | NA | NA | p.I209I p.P156P | p.E62K p.R469I | p.E1446* p.R269Q p.R733Q | NA |
| TCGA-DF-A2KV | NA | NA | NA | NA | NA | p.L261M | NA | NA |
| TCGA-DI-A1BU | NA | NA | NA | p.V302A | NA | NA | p.G1108G | NA |
| TCGA-FI-A2D0 | NA | NA | p.I283V | p.T119T | p.A457T | p.E477D | p.E830G | NA |
| TCGA-QS-A5YQ | NA | NA | NA | NA | NA | NA | NA | NA |
| TCGA-AJ-A5DW | NA | NA | NA | NA | p.E70* | NA | NA | NA |
| TCGA-AX-A2HG | NA | NA | NA | p.X314_splice | NA | NA | NA | NA |
| TCGA-BS-A0V4 | NA | NA | NA | NA | NA | NA | NA | NA |
| TCGA-D1-A16Y | NA | NA | NA | NA | NA | NA | NA | NA |
| TCGA-EO-A22R | p.R393R | p.R473W | NA | NA | p.R226W p.T120T | p.X273_splice | p.K705K p.S1448R | p.L243R |
| TCGA-EO-A22T | NA | NA | NA | NA | NA | NA | NA | NA |
| TCGA-FI-A2D5 | NA | NA | p.R12H | p.K538N | p.A454V p.N3S p.R256H p.R597C | NA | p.E91G p.H643R p.I1326V p.R1032H | p.E40E p.R64C |
| TCGA-D1-A0ZR | NA | NA | NA | NA | NA | NA | NA | p.K65* p.R298P |
| TCGA-EC-A24G | NA | NA | NA | NA | NA | NA | NA | NA |
| TCGA-QF-A5YS | NA | NA | NA | p.F543L | NA | p.S357Y | NA | NA |
| TCGA-AJ-A8CT | NA | NA | NA | NA | NA | NA | p.R1756* p.R454* | NA |
| TCGA-AX-A3G3 | NA | NA | NA | NA | NA | NA | p.S617S | NA |
| TCGA-BS-A0TA | NA | NA | NA | NA | NA | NA | NA | NA |
| TCGA-EO-A3AY | NA | NA | NA | NA | NA | NA | p.A519V p.D1489G p.F31C p.L1099I | p.E49* |
| TCGA-AP-A1DR | NA | NA | NA | NA | NA | NA | NA | NA |
| TCGA-AP-A1DV | NA | p.C338C p.R473W | NA | NA | p.I310I p.S81N | NA | p.Q766H p.R130I p.T655T | NA |
| TCGA-B5-A11Y | NA | NA | NA | NA | NA | NA | NA | NA |
| TCGA-AX-A1CE | NA | p.R337H p.S482L | p.G77G | NA | NA | p.A255T p.N532N | NA | p.A172A |
| TCGA-BG-A0MA | NA | NA | NA | NA | NA | NA | NA | NA |
| TCGA-D1-A101 | NA | NA | NA | NA | NA | NA | NA | NA |
| TCGA-D1-A1NZ | NA | NA | NA | NA | NA | NA | NA | NA |
| TCGA-D1-A1O7 | NA | NA | NA | NA | p.X60_splice | NA | NA | NA |
| TCGA-DF-A2L0 | NA | NA | NA | NA | NA | NA | NA | NA |
| TCGA-EY-A215 | p.R392W | NA | NA | NA | NA | NA | p.V1204I | NA |
| TCGA-D1-A17H | p.R358W | NA | NA | NA | NA | NA | NA | NA |
| TCGA-QS-A5YR | NA | NA | NA | NA | NA | NA | p.R1640H | NA |
| TCGA-BG-A3EW | NA | NA | NA | NA | NA | NA | NA | NA |
| TCGA-AX-A2IN | NA | p.A437V | NA | NA | NA | NA | NA | NA |
| TCGA-AX-A3G9 | NA | NA | NA | p.R167C | NA | NA | NA | NA |
| TCGA-AX-A3FT | NA | NA | p.P114P | NA | NA | NA | NA | NA |
| TCGA-B5-A11J | NA | NA | NA | p.R199Q | NA | NA | p.R1007C | NA |
| TCGA-FI-A2F4 | NA | NA | NA | NA | NA | NA | NA | NA |
| TCGA-SJ-A6ZI | NA | NA | NA | NA | NA | NA | NA | NA |
| TCGA-AJ-A3BG | NA | NA | NA | NA | NA | NA | NA | NA |
| TCGA-B5-A1MR | NA | NA | p.S38L | NA | NA | p.S438S | p.D251Y p.R454* p.R568I | NA |
| TCGA-BS-A0UF | NA | p.L51I | p.S38L | NA | p.A337V p.K151N | NA | p.E1097* p.Q1171Q p.R446C p.S1367Y | p.R354* |
| TCGA-EY-A1GO | NA | NA | NA | NA | NA | NA | NA | NA |
| TCGA-EY-A1GU | NA | NA | NA | NA | NA | NA | NA | NA |
| TCGA-A5-A0G1 | NA | NA | NA | NA | NA | p.P89L p.S10N | NA | p.K273K p.R52M |
| TCGA-B5-A11H | NA | NA | NA | NA | NA | NA | p.F721Lfs*11 | NA |
| TCGA-A5-A0RA | NA | NA | NA | p.I83T | NA | NA | NA | NA |
| TCGA-A5-A2K3 | NA | NA | NA | NA | NA | NA | NA | NA |
| TCGA-BG-A0RY | NA | NA | NA | NA | NA | NA | NA | p.R298P |
| TCGA-BS-A0UJ | NA | NA | NA | NA | NA | NA | NA | NA |
| TCGA-EO-A22Y | NA | NA | NA | NA | NA | NA | NA | NA |
| TCGA-PG-A6IB | NA | NA | NA | NA | NA | NA | NA | NA |
| TCGA-AJ-A23O | NA | NA | NA | NA | NA | NA | NA | NA |
| TCGA-AP-A059 | p.S384N | NA | NA | p.Q409Q | NA | p.G422D | p.R1793I | NA |
| TCGA-AX-A2HD | NA | p.A286V | NA | NA | p.L378L | p.T405T | p.D329D p.E1133D p.R1052H p.R446H p.R658* | NA |
| TCGA-B5-A11N | NA | NA | NA | NA | p.H413N | NA | NA | NA |
| TCGA-BG-A220 | NA | NA | NA | NA | NA | NA | p.V1267V | NA |
| TCGA-BS-A0TC | NA | NA | NA | NA | NA | p.K473T | NA | NA |
| TCGA-AP-A1DP | NA | NA | NA | NA | NA | NA | NA | NA |
| TCGA-D1-A167 | NA | NA | NA | p.P459P | NA | NA | p.T655M | NA |
| TCGA-D1-A177 | NA | NA | NA | NA | NA | NA | NA | p.S299_T300del |
| TCGA-D1-A2G0 | NA | NA | NA | NA | NA | NA | p.S1366Vfs*3 | NA |
| TCGA-B5-A5OC | NA | NA | NA | p.K228E | NA | NA | NA | NA |
| TCGA-BG-A0MQ | NA | NA | p.G256Vfs*27 | NA | NA | NA | p.R1768W | NA |
| TCGA-BK-A26L | NA | NA | NA | NA | NA | NA | NA | NA |
| TCGA-EC-A1QX | NA | NA | NA | NA | NA | NA | NA | NA |
| TCGA-EO-A3AU | NA | NA | NA | NA | NA | NA | NA | NA |
| TCGA-AJ-A3OK | NA | NA | NA | NA | NA | NA | NA | NA |
| TCGA-AX-A1C9 | NA | p.R80W | NA | p.T273M | NA | NA | NA | NA |
| TCGA-B5-A3FA | NA | NA | NA | p.R551Q | p.E506E | p.G425G | p.A758T p.M722Yfs*5 p.N598D p.R1178W p.S845T p.V76A | p.S399L |
| TCGA-EC-A1NJ | NA | NA | NA | NA | NA | NA | p.H1596H | NA |
| TCGA-B5-A11U | NA | NA | NA | NA | NA | NA | p.F721Lfs*11 | NA |
| TCGA-EY-A1GK | NA | NA | NA | NA | p.H181H | NA | NA | NA |
| TCGA-FI-A2D6 | NA | NA | NA | NA | NA | NA | NA | NA |
| TCGA-PG-A917 | NA | NA | NA | p.A339A | NA | NA | NA | NA |
| TCGA-D1-A0ZO | NA | NA | NA | NA | NA | NA | NA | NA |
| TCGA-A5-A2K5 | NA | NA | NA | NA | NA | NA | p.F559L | p.R456Q |
| TCGA-AX-A3G8 | NA | NA | NA | NA | NA | NA | NA | NA |
| TCGA-E6-A1LZ | NA | NA | NA | NA | NA | NA | NA | NA |
| TCGA-AX-A1C7 | NA | NA | NA | NA | NA | NA | p.K1388K | NA |
| TCGA-SL-A6JA | NA | NA | NA | NA | NA | NA | p.S1366Vfs*3 | NA |
| TCGA-EY-A549 | NA | NA | NA | NA | NA | NA | NA | NA |
| TCGA-D1-A0ZZ | NA | p.K2Nfs*9 | NA | NA | NA | NA | NA | NA |
| TCGA-EO-A3AZ | NA | p.A286V | NA | NA | NA | NA | NA | NA |
| TCGA-EO-A3KX | NA | NA | NA | NA | NA | NA | NA | NA |
| TCGA-D1-A17L | NA | NA | NA | NA | NA | NA | NA | p.R298P |
| TCGA-AX-A3FS | NA | p.R445H | NA | NA | NA | NA | NA | NA |
| TCGA-EO-A22U | p.S300S | NA | p.R167Q | p.A40T p.E116K p.R575Q | NA | p.A237V p.L97M | NA | NA |
| TCGA-EY-A1G8 | NA | NA | NA | NA | NA | NA | NA | p.L152F p.R228Q |
| TCGA-EY-A548 | NA | NA | NA | NA | NA | NA | NA | NA |
| TCGA-BG-A221 | NA | NA | NA | p.A283V p.V410A | NA | NA | NA | NA |
| TCGA-AJ-A2QO | NA | NA | NA | NA | NA | NA | NA | NA |
| TCGA-AX-A2H2 | NA | NA | NA | p.P524Q | NA | NA | NA | NA |
| TCGA-D1-A103 | NA | NA | p.R12R | p.G212D | p.T479I p.T531N | NA | p.S1787S | NA |
| TCGA-DF-A2KZ | NA | NA | NA | NA | NA | NA | p.S1111Lfs*51 | NA |
| TCGA-BG-A0W1 | NA | NA | NA | NA | NA | NA | NA | NA |
| TCGA-DF-A2KN | NA | NA | NA | p.P250P | p.T531T | p.A444T | NA | NA |
| TCGA-DF-A2KU | NA | p.A311A | p.R167* | NA | p.T215I | p.D137G p.E262D p.R171H p.T405T | NA | p.C51C p.S399L |

| **UCEC sampleID** | **METTL3** | **RBM15** | **WTAP** | **YTHDC1** | **YTHDC2** | **YTHDF1** | **YTHDF2** | **YTHDF3** | **ZC3H13** |
| --- | --- | --- | --- | --- | --- | --- | --- | --- | --- |
| TCGA-AP-A0LM | NA | NA | NA | p.E25V p.R59Q | p.A33V p.R911Q | p.A128A | p.I129V | p.A164D p.W410* | p.K233E p.R1158K |
| TCGA-BK-A6W3 | NA | p.D934A | NA | NA | p.E185K p.K1200T | NA | NA | NA | p.R1216Q |
| TCGA-AX-A2HA | NA | NA | NA | NA | p.V976M | NA | NA | NA | p.K1006Nfs*31 |
| TCGA-EO-A22X | p.I378I | p.E111D p.S866R | NA | p.R267Q p.R51Q | p.N718H p.S347Y p.T271A | p.P379P | NA | NA | p.R725C |
| TCGA-AP-A1E0 | NA | NA | NA | NA | p.R430I | NA | NA | NA | p.S1534Y |
| TCGA-E6-A1LX | NA | NA | NA | p.D541A p.E12* p.E124K p.R451C p.R715* | p.E201* p.R1002* p.R1247Q p.V961I | NA | NA | NA | p.K313T p.R1304* p.R646Q |
| TCGA-A5-A2K7 | NA | NA | NA | NA | NA | NA | NA | NA | NA |
| TCGA-EO-A3B0 | NA | p.N796S p.S130N | p.P8H | NA | p.E634K p.I205L | p.S50S | p.E519K | p.R352H | NA |
| TCGA-A5-A1OF | p.D88E p.P371H | p.A862D p.R456H | p.A159V | p.D541V p.R338C p.T379T | NA | NA | p.P332H p.Q325R p.T217A | NA | p.R1341* p.R933C |
| TCGA-AJ-A3EL | NA | NA | NA | NA | p.A977T p.R265Q | NA | NA | NA | NA |
| TCGA-AX-A0J0 | p.I503I p.L580V | p.K203N | NA | p.K332Q p.R341Q | p.E185K p.E393* p.E966D p.N1196T | NA | NA | NA | p.L1563V p.R640I |
| TCGA-AX-A1C5 | NA | NA | NA | p.R536Q | NA | NA | NA | NA | p.R797* p.S380L |
| TCGA-B5-A1MX | NA | p.P645P | p.X203_splice | NA | NA | NA | NA | p.P116Q | NA |
| TCGA-BG-A222 | NA | NA | NA | NA | NA | NA | NA | NA | NA |
| TCGA-B5-A0JY | NA | p.K445T | p.R374Q | NA | p.R98K p.X93_splice | NA | NA | p.D407Y | p.R1341* |
| TCGA-BG-A0M8 | NA | NA | NA | NA | NA | NA | NA | NA | NA |
| TCGA-D1-A163 | NA | NA | NA | NA | NA | p.V502A | NA | NA | NA |
| TCGA-D1-A16N | NA | NA | NA | p.G600G | NA | NA | NA | NA | NA |
| TCGA-D1-A174 | NA | NA | p.Q66Q | NA | p.M844Wfs*8 | NA | NA | NA | NA |
| TCGA-EO-A3AV | NA | NA | NA | NA | p.E201* p.E966D | NA | NA | NA | p.R552Q |
| TCGA-EY-A1GD | NA | NA | NA | NA | NA | NA | NA | NA | NA |
| TCGA-AX-A1C4 | NA | NA | NA | NA | NA | NA | NA | NA | NA |
| TCGA-BG-A0MK | NA | NA | NA | NA | NA | NA | NA | p.A73P | NA |
| TCGA-EY-A1GI | NA | NA | NA | NA | p.E634K | p.E363K | NA | NA | p.R1284* |
| TCGA-AJ-A3EK | NA | NA | NA | p.K651E | p.T1056T | p.R556Q | p.G362S p.Q338H | p.P267P | NA |
| TCGA-AP-A1DK | p.D138D p.S64G | NA | p.K312E | p.Q68H | p.G107* p.S91N | p.G459D p.P324P p.Q308Q | NA | NA | p.R170C |
| TCGA-AX-A2HC | p.K578N | NA | NA | p.D282Y p.R414I | p.Q552H p.Q758H p.R1216K p.S1378N | NA | NA | p.R519H | p.R559Q |
| TCGA-B5-A11E | p.S310Y | p.T882T | NA | NA | NA | NA | NA | p.A210V | p.S1240I p.S325F |
| TCGA-B5-A3FC | NA | NA | p.A159V | p.N196D | p.R242H | p.N335N p.R506W p.S346S | NA | p.S23S | NA |
| TCGA-BG-A18B | NA | NA | NA | NA | NA | NA | NA | NA | NA |
| TCGA-E6-A2P8 | NA | NA | NA | p.V70A | NA | NA | NA | NA | NA |
| TCGA-AJ-A3OJ | NA | NA | NA | NA | NA | NA | NA | NA | NA |
| TCGA-D1-A16X | p.S511S | NA | NA | NA | p.L1115M | p.I523I | NA | p.D509Y | p.G816E |
| TCGA-D1-A17Q | p.R471H p.S184L | p.E609K | p.L119V | p.E300D p.K361N p.R695Q | p.E149K p.E217* p.K708T | NA | NA | NA | p.R946* |
| TCGA-B5-A1MW | NA | NA | NA | NA | NA | NA | NA | NA | NA |
| TCGA-BS-A0U8 | NA | NA | NA | NA | NA | NA | NA | NA | p.R1302* |
| TCGA-BS-A0UV | p.E442* | NA | NA | p.E124* p.R154I | NA | p.D31Y | NA | p.Q141R | p.D732Y p.S1014F |
| TCGA-DF-A2KV | NA | NA | NA | NA | NA | NA | NA | NA | NA |
| TCGA-DI-A1BU | NA | NA | NA | p.R51Q p.S71S | p.S753S p.S82S p.V1062A p.Y595H | NA | NA | NA | NA |
| TCGA-FI-A2D0 | NA | NA | p.A116V p.M124Nfs*4 | p.R525C | p.E634K | NA | NA | p.R516R | p.R493C p.R842C |
| TCGA-QS-A5YQ | NA | NA | NA | NA | p.E767K | NA | NA | NA | p.R725S |
| TCGA-AJ-A5DW | NA | p.K87N | NA | p.A373V | p.D334Y p.D705Y p.L925I | NA | NA | NA | NA |
| TCGA-AX-A2HG | NA | NA | NA | NA | NA | NA | NA | NA | NA |
| TCGA-BS-A0V4 | NA | NA | NA | NA | NA | NA | NA | NA | p.R693Gfs*3 |
| TCGA-D1-A16Y | NA | NA | NA | NA | p.F599F | NA | NA | NA | p.K14N |
| TCGA-EO-A22R | p.G60G p.R133Q p.T348T | p.D242Y | p.R79* | p.E172K p.X608_splice | p.A262T p.E1164K | p.F99I | p.G282D | p.P127Sfs*3 p.R452H | p.K310N p.R1513* p.R396* p.R607I p.R904C |
| TCGA-EO-A22T | NA | NA | NA | NA | NA | NA | NA | NA | p.S1660N |
| TCGA-FI-A2D5 | NA | p.Y251H | NA | p.P641P p.R685Q p.S435N | p.D1091D p.L773L p.N122N | p.G13* | NA | p.G90G | p.R1267C p.R1304* p.R477W p.S1098F |
| TCGA-D1-A0ZR | NA | NA | NA | NA | NA | NA | NA | NA | NA |
| TCGA-EC-A24G | NA | p.V200I | NA | NA | NA | NA | NA | NA | NA |
| TCGA-QF-A5YS | NA | NA | NA | NA | NA | NA | NA | NA | p.T541M |
| TCGA-AJ-A8CT | NA | NA | NA | NA | NA | NA | NA | NA | NA |
| TCGA-AX-A3G3 | NA | NA | NA | NA | NA | NA | NA | NA | NA |
| TCGA-BS-A0TA | NA | NA | NA | NA | NA | NA | p.V284I | NA | NA |
| TCGA-EO-A3AY | NA | NA | NA | NA | NA | NA | NA | NA | NA |
| TCGA-AP-A1DR | NA | NA | NA | NA | NA | p.E125* | p.D504D | NA | NA |
| TCGA-AP-A1DV | NA | NA | p.K9T | p.R666H p.S164N | p.K1278T p.K781K | p.A337A | p.R447H | p.G332E | p.E802* p.T1150T |
| TCGA-B5-A11Y | p.R508H | NA | NA | p.R451C | NA | NA | NA | NA | NA |
| TCGA-AX-A1CE | NA | p.G650* | p.S313S p.T148T | p.G702R p.Q624H p.R116C | p.K852K p.R888H p.T330I p.X225_splice | NA | NA | NA | p.R1349* p.R725H p.R933H |
| TCGA-BG-A0MA | NA | NA | NA | NA | p.L566P | NA | NA | NA | NA |
| TCGA-D1-A101 | NA | NA | NA | NA | NA | NA | NA | p.R357H | NA |
| TCGA-D1-A1NZ | NA | NA | NA | NA | NA | NA | NA | NA | p.G997Rfs*8 |
| TCGA-D1-A1O7 | NA | NA | NA | NA | NA | NA | NA | NA | p.R1007H |
| TCGA-DF-A2L0 | NA | p.R618R | NA | NA | NA | NA | NA | NA | NA |
| TCGA-EY-A215 | NA | NA | NA | NA | NA | p.A337A p.A424T | NA | NA | p.R1311C |
| TCGA-D1-A17H | NA | NA | NA | NA | NA | NA | NA | NA | NA |
| TCGA-QS-A5YR | NA | NA | NA | NA | NA | NA | p.P268Rfs*2 | NA | NA |
| TCGA-BG-A3EW | NA | NA | NA | p.R525H | NA | NA | NA | NA | NA |
| TCGA-AX-A2IN | NA | NA | NA | p.R699* | NA | NA | NA | NA | NA |
| TCGA-AX-A3G9 | NA | NA | NA | NA | NA | NA | NA | NA | NA |
| TCGA-AX-A3FT | p.H330R | NA | NA | NA | NA | NA | NA | NA | NA |
| TCGA-B5-A11J | NA | NA | NA | NA | NA | NA | NA | NA | NA |
| TCGA-FI-A2F4 | NA | p.H545H | NA | p.E249del | NA | NA | NA | p.Q584Q | NA |
| TCGA-SJ-A6ZI | NA | NA | NA | NA | NA | NA | NA | NA | p.L1470V |
| TCGA-AJ-A3BG | p.R267* | NA | NA | NA | NA | p.R420C | NA | NA | NA |
| TCGA-B5-A1MR | NA | NA | p.R79* | p.R715* | p.A96T p.E1163D | NA | NA | p.R581I | p.R369H p.R725C p.R811R |
| TCGA-BS-A0UF | NA | p.N133H | NA | NA | p.E263D p.E711* p.X1404_splice | p.V104V | NA | NA | p.R453Q p.S600R |
| TCGA-EY-A1GO | NA | NA | NA | NA | NA | NA | NA | p.L70F | NA |
| TCGA-EY-A1GU | NA | NA | NA | NA | NA | NA | NA | NA | p.R693Gfs*3 |
| TCGA-A5-A0G1 | p.Q121H | NA | NA | p.F391F p.P537T p.V503A | p.A537V p.T1313S | NA | p.G13C | NA | NA |
| TCGA-B5-A11H | NA | NA | NA | NA | NA | NA | NA | NA | NA |
| TCGA-A5-A0RA | NA | NA | NA | NA | NA | NA | NA | NA | NA |
| TCGA-A5-A2K3 | NA | NA | NA | NA | NA | NA | NA | NA | p.K970R |
| TCGA-BG-A0RY | NA | NA | NA | NA | NA | NA | NA | NA | NA |
| TCGA-BS-A0UJ | NA | NA | NA | p.D566V | p.I1017I | NA | NA | NA | NA |
| TCGA-EO-A22Y | NA | NA | p.R162P | NA | NA | NA | NA | NA | NA |
| TCGA-PG-A6IB | NA | p.R57L | NA | NA | NA | NA | NA | NA | NA |
| TCGA-AJ-A23O | NA | NA | NA | NA | NA | NA | NA | NA | p.D638Sfs*14 |
| TCGA-AP-A059 | NA | NA | NA | p.E178K p.E259* | p.E185K p.R80Q p.S1166Y | p.V391V | p.N397I | NA | p.D1413A p.R103* p.R903R |
| TCGA-AX-A2HD | NA | NA | NA | NA | NA | NA | p.K178K | NA | p.G997Dfs*40 p.R93H |
| TCGA-B5-A11N | NA | NA | NA | NA | NA | NA | NA | NA | NA |
| TCGA-BG-A220 | NA | NA | NA | NA | p.N1427Kfs*4 | NA | NA | NA | NA |
| TCGA-BS-A0TC | NA | NA | NA | NA | p.E185K | NA | NA | NA | NA |
| TCGA-AP-A1DP | NA | p.R473G | NA | NA | NA | NA | NA | NA | NA |
| TCGA-D1-A167 | NA | p.R729K | NA | NA | NA | NA | NA | NA | NA |
| TCGA-D1-A177 | NA | NA | NA | NA | NA | NA | NA | NA | p.X574_splice |
| TCGA-D1-A2G0 | NA | NA | NA | NA | NA | NA | NA | NA | NA |
| TCGA-B5-A5OC | NA | NA | NA | NA | NA | NA | NA | NA | p.R562* |
| TCGA-BG-A0MQ | NA | NA | NA | NA | NA | NA | NA | NA | NA |
| TCGA-BK-A26L | NA | NA | NA | NA | p.S652C | NA | NA | NA | NA |
| TCGA-EC-A1QX | NA | p.G138Afs*35 | NA | p.R674W | p.T454S | NA | NA | NA | p.R1349Nfs*15 |
| TCGA-EO-A3AU | NA | NA | NA | NA | NA | NA | NA | p.A486V | NA |
| TCGA-AJ-A3OK | NA | NA | NA | p.R525C | NA | NA | NA | NA | NA |
| TCGA-AX-A1C9 | NA | p.Y608Y | NA | NA | NA | NA | NA | NA | NA |
| TCGA-B5-A3FA | NA | p.A552V | p.R79* | p.D495N p.R703R p.V503A | p.D669N p.I1046M p.I1303I p.K887R p.T166I p.T390A | NA | p.D422N p.G21* p.G453G | p.G470G | p.D1013D p.D1354D p.G1426E p.R618R p.T1139I |
| TCGA-EC-A1NJ | NA | NA | NA | NA | NA | NA | NA | NA | NA |
| TCGA-B5-A11U | NA | NA | NA | NA | NA | NA | NA | NA | NA |
| TCGA-EY-A1GK | NA | NA | NA | NA | NA | NA | NA | NA | p.R741Gfs*41 |
| TCGA-FI-A2D6 | NA | NA | NA | NA | NA | NA | NA | p.S144S | NA |
| TCGA-PG-A917 | NA | NA | NA | NA | NA | NA | p.R355R | NA | p.Y522H |
| TCGA-D1-A0ZO | NA | NA | p.F147S | NA | NA | NA | NA | NA | NA |
| TCGA-A5-A2K5 | p.R179H | NA | NA | NA | NA | NA | NA | NA | p.R531Q |
| TCGA-AX-A3G8 | NA | NA | NA | NA | p.A261T | NA | NA | NA | NA |
| TCGA-E6-A1LZ | NA | NA | NA | NA | NA | NA | p.S113C | NA | NA |
| TCGA-AX-A1C7 | NA | NA | NA | NA | NA | NA | NA | NA | NA |
| TCGA-SL-A6JA | NA | NA | NA | NA | NA | NA | NA | NA | NA |
| TCGA-EY-A549 | NA | p.P284Lfs*98 | NA | NA | NA | NA | NA | NA | p.K279Nfs*8 |
| TCGA-D1-A0ZZ | NA | NA | NA | NA | NA | NA | NA | NA | NA |
| TCGA-EO-A3AZ | NA | NA | NA | NA | NA | NA | NA | NA | p.A777V |
| TCGA-EO-A3KX | NA | p.E363G | NA | p.E249del | NA | NA | NA | NA | p.D1401G |
| TCGA-D1-A17L | NA | NA | NA | NA | NA | NA | NA | NA | NA |
| TCGA-AX-A3FS | NA | NA | NA | p.E223G | NA | NA | NA | NA | NA |
| TCGA-EO-A22U | p.A55T | p.A503A p.C667Y p.D101N p.P334L p.R661H p.R963R | p.A159V | p.A373A p.E124K p.G422R p.R525H p.R674Q | p.R1247Q | NA | p.P265P p.R357H p.S138N | p.R446H | NA |
| TCGA-EY-A1G8 | NA | NA | NA | NA | NA | p.V206V | NA | NA | NA |
| TCGA-EY-A548 | NA | NA | p.R162P | NA | NA | NA | NA | NA | NA |
| TCGA-BG-A221 | NA | NA | NA | NA | NA | NA | NA | NA | p.R681W |
| TCGA-AJ-A2QO | NA | NA | NA | NA | NA | NA | p.R494H | NA | NA |
| TCGA-AX-A2H2 | NA | NA | NA | NA | NA | NA | NA | NA | NA |
| TCGA-D1-A103 | NA | NA | p.P103S | NA | p.A928T p.L626L p.Q774Q | p.N16T | NA | NA | p.E1472* |
| TCGA-DF-A2KZ | NA | NA | NA | NA | NA | NA | NA | NA | p.D1408D |
| TCGA-BG-A0W1 | NA | NA | NA | NA | NA | p.V207I | NA | NA | NA |
| TCGA-DF-A2KN | NA | NA | p.T321I | NA | NA | NA | p.E420D | NA | p.A1517Lfs*4 p.R1481M |
| TCGA-DF-A2KU | p.A287V | p.D264Y p.R963K | NA | NA | p.A740T p.D443N p.G581E p.K37N p.R1002Q | NA | NA | p.G182D p.K281Q | p.L1421M |
